# Abnormal Dynamic Functional Connectivity Within the Fronto‐Limbic Network Mediates the Association Between Depressive Symptoms and Non‐Suicidal Self‐Injury in Adolescents

**DOI:** 10.1002/cns.71023

**Published:** 2026-07-21

**Authors:** Yongjie Zhou, Shengmiao Ni, Junrong Han, Binke Yuan, Xinni Luo, Haiyan Li, Nan Yan, Lan Wang, Wei Zhang, Lingyun Zeng, Xuchu Weng, Lijuan Huo

**Affiliations:** ^1^ Shenzhen Maternity and Child Healthcare Hospital, Southern Medical University Shenzhen China; ^2^ Key Laboratory of Brain, Cognition and Education Science, Ministry of Education; Institute for Brain Research and Rehabilitation, and Guangdong Key Laboratory of Mental Health and Cognitive Science South China Normal University Guangzhou China; ^3^ Department of Psychiatry The Affiliated Brain Hospital of Guangzhou Medical University Guangzhou China; ^4^ CAS Key Laboratory of Human‐Machine Intelligence‐Synergy Systems, Shenzhen Institutes of Advanced Technology Chinese Academy of Sciences Shenzhen China; ^5^ Shenzhen Kangning Hospital Shenzhen China

**Keywords:** adolescents, depression, dynamic functional connectivity, machine learning, non‐suicidal self‐injury, the fronto‐limbic network

## Abstract

**Aim:**

Non‐suicidal self‐injury (NSSI) is a prevalent behavior among adolescents with major depressive disorder (MDD), yet the precise neural mechanisms remain unclear. This study aimed to investigate the temporal dynamics of brain connectivity associated with NSSI in adolescents with MDD using dynamic functional connectivity (dFC) analysis.

**Methods:**

Resting‐state fMRI data from 204 adolescents (154 with NSSI, 50 without) were analyzed. dFC variability within the fronto‐limbic network was assessed using a seed‐based dynamic conditional correlation approach. Group differences in dFC variability were examined, and a machine‐learning model was used to predict NSSI based on dFC features. Mediation analysis explored the dFC's role in the relationship between depressive symptoms and NSSI.

**Results:**

Adolescents with NSSI exhibited reduced dFC variability, which mediated the relationship between depressive severity and NSSI behavior (a*b = 0.144; *p* = 0.001). Key connections—insula, anterior cingulate cortex, orbitofrontal cortex, and hippocampus—were critical in distinguishing NSSI from non‐NSSI groups. Machine learning models based on these connections achieved robust and stable performance with mean AUC of 0.84 and PR‐AUC of 0.94 in predicting NSSI.

**Conclusions:**

Altered dFC within the fronto‐limbic network may underlie NSSI in adolescents with MDD, identifying preliminary neural features for targeted interventions and highlighting neurobiological heterogeneity associated with NSSI in adolescents with MDD.

## Graphical abstract

This study systematically investigated the abnormalities of dynamic functional connectivity within the fronto‐limbic network in depressed adolescents with non‐suicidal self‐injury (NSSI), and the relationship between these abnormalities, depressive symptoms, and NSSI behavior. These findings uncover the potential neurobiological mechanism and therapeutic target of NSSI.

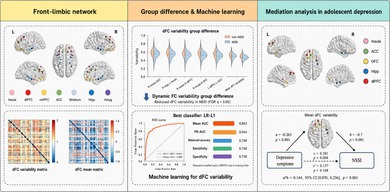

## 1 Introduction

Non‐suicidal self‐injury (NSSI) refers to deliberate behaviors, such as hitting, head banging, or cutting, to inflict harm upon oneself without any suicidal thoughts [1]. It is a common and clinically significant behavior in adolescence, particularly among individuals with major depressive disorder (MDD) [2, 3, 4, 5, 6]. Depressed adolescents showed more NSSI engagement than adolescents without depression [4, 7]. However, most previous studies have focused on community samples, rather than adolescents with MDD. Longitudinal studies have shown that depressive symptoms predict higher frequencies and longer persistence of NSSI in the future [8, 9, 10]. NSSI is also widely conceptualized as a maladaptive strategy for regulating overwhelming negative affect or emotional pain [11, 12, 13]. Together, depressive symptoms are closely associated with NSSI but the specific neural mechanisms underlying this association, particularly in adolescents with MDD, remain insufficiently understood.

Converging evidence implicates the fronto‐limbic network in the neurobiology of NSSI [14, 15], including the prefrontal cortex (PFC), hippocampus, striatum, amygdala, and anterior cingulate cortex (ACC) [16, 17]. The amygdala processes emotional stimuli while the PFC regulates impulse control and decision‐making, dysfunction of this connectivity leading to impulsive behaviors, including NSSI [14, 18]. Additionally, the PFC modulates the pain system and reward system, such as the ACC, basal nuclei, and thalamus [19]. The PFC has been a critical brain stimulation target for effectively treating depression and NSSI [20, 21]. These findings indicate the role of the fronto‐limbic network in emotional perception and regulation, impulse control, stress processing, and pain perception—functions that are highly relevant to self‐injurious behavior.

Aberrant functional connectivity (FC) within the fronto‐limbic network has been associated with NSSI, suggesting that dysfunction in this circuitry may be relevant to the neural basis of self‐injurious behavior. Compared with healthy controls, adolescents with NSSI have aberrant FC between the PFC and ACC, amygdala, or insula [22, 23, 24]. However, these findings have been inconsistent, with both hypo‐ and hyper‐connectivity reported across studies. One possible reason is that most previous work has relied on static functional connectivity, which assumes that connectivity remains stable throughout the scan and may therefore overlook transient but meaningful fluctuations in neural interactions [25]. By contrast, dynamic FC (dFC) captures time‐varying changes in connectivity, providing a framework to characterize how interactions between brain regions evolve over time [26]. This temporal perspective may be particularly relevant to NSSI, as self‐injurious behavior is often preceded by rapidly fluctuating emotional distress and urges [27, 28], processes that are closely linked to fronto‐limbic network function. Accordingly, dFC may offer a more sensitive approach for detecting neural alterations underlying NSSI. To our knowledge, this approach has rarely been applied to adolescents with MDD who engage in NSSI.

To investigate the questions mentioned before, the current study applies a dynamic conditional correlation (DCC) approach to examine dFC variability within the fronto‐limbic network in adolescents with MDD. Employing a clinical control group comprising adolescents with MDD who do not engage in NSSI allows for the differentiation of neurobiological patterns specific to self‐injurious behavior from general features of depressive pathology. This study has two aims: (1) to identify specific alterations in dFC variability within the fronto‐limbic network associated with NSSI, (2) to examine whether altered dFC is related to depressive symptom severity and mediates the relationship between severity of depressive symptoms and NSSI behavior.

## 2 Methods

### 2.1 Participants and Clinical Assessment

A total of 234 participants were enrolled in Shenzhen Kangning Hospital. Inclusion criteria were: (1) diagnosed with MDD by two experienced clinical psychiatrists based on the criteria of the Diagnostic and Statistical Manual of Mental Disorders, Fifth Edition (DSM‐V); (2) aged from 12 to 18 years old; (3) right‐handedness. Exclusion criteria included: (1) other neurological illness or intellectual disability; (2) comorbidity with bipolar depression, substance use disorders, or other mental disorders based on DSM‐V diagnosed by two experienced clinical psychiatrists during the outpatient period; (3) MRI contraindications.

All participants completed the self‐made questionnaire with the assistance of graduate students to collect demographic and clinical information such as age, gender, substance use, family history of mental illness. The clinical assessment comprised of three scales: Functional Assessment of Self‐Mutilation (FASM), Patient Health Questionnaire (PHQ‐9), and Generalized Anxiety Disorder Scale (GAD‐7). The details of the scales and assessment procedures are provided in Appendix S1.

After data quality control, one participant was excluded due to excessive head motion during MRI acquisition, and 29 participants were excluded because of incomplete questionnaire data. The specific enrollment process of all participants and procedure of quality control detailed in Appendix S1 and Figure S1.

### 2.2 MRI Data Acquisition and Pre‐Processing

MRI data were obtained using a 3.0 Tesla scanner MRI (Prisma, Siemens, Germany). During the scanning, participants were instructed on minimizing movement and provided with earplugs to reduce scanner noise discomfort, and were asked to remain still and awake while allowing their thoughts to flow naturally. High‐resolution structural images were acquired with a standard FSPGR T1‐weighted sequence. The functional images were acquired using an echo‐planar imaging (EPI) sequence. The functional images were preprocessed by Analysis of Functional Neuro Images software (AFNI v 23.1.00 [29]). Preprocessing included slice timing correction, head motion correction, realignment, standardization, smoothing, and bandpass filtering. Details of preprocessing are provided in Appendix S1. To minimize motion confounds, index of head motion, and their temporal derivatives, were regressed out. The index of head motion in this research was Framewise displacement (FD). The participants with a mean FD > 0.2 mm were excluded.

### 2.3 Dynamic Functional Connectivity Analysis

Using the preprocessed fMRI data, we constructed a dynamic functional network with the seed‐based DCC approach [30] with MATLAB R2022a. The DCC method has demonstrated higher sensitivity and specificity in detecting these dynamic changes [30]. The computation of DCC and the visualization of results were performed using the NaDyNet toolbox [31], available at [https://github.com/yuanbinke/Naturalistic‐Dynamic‐Network‐Toolbox]. We estimated dFC between all pairs of 55 regions of interest (ROI) for each time point of every participant (see Figure S2 for visual representation and Table S1 for ROI coordinates in Supporting Information). Of these, 47 ROIs were subregions within the fronto‐limbic network, delineated based on a large 268‐node functional brain atlas [32], including the bilateral amygdala, striatum, ACC, hippocampus, dorsolateral PFC (dlPFC) and ventromedial PFC (vmPFC). Additionally, considering that the anterior insula has been described as a paralimbic structure [33], crucial for pain processing and relevant to NSSI behavior [34], 8 ROIs were extracted from the anterior insula, including 4 subdivisions from both right and left hemispheres, delineated in the same atlas.

**FIGURE 1:**
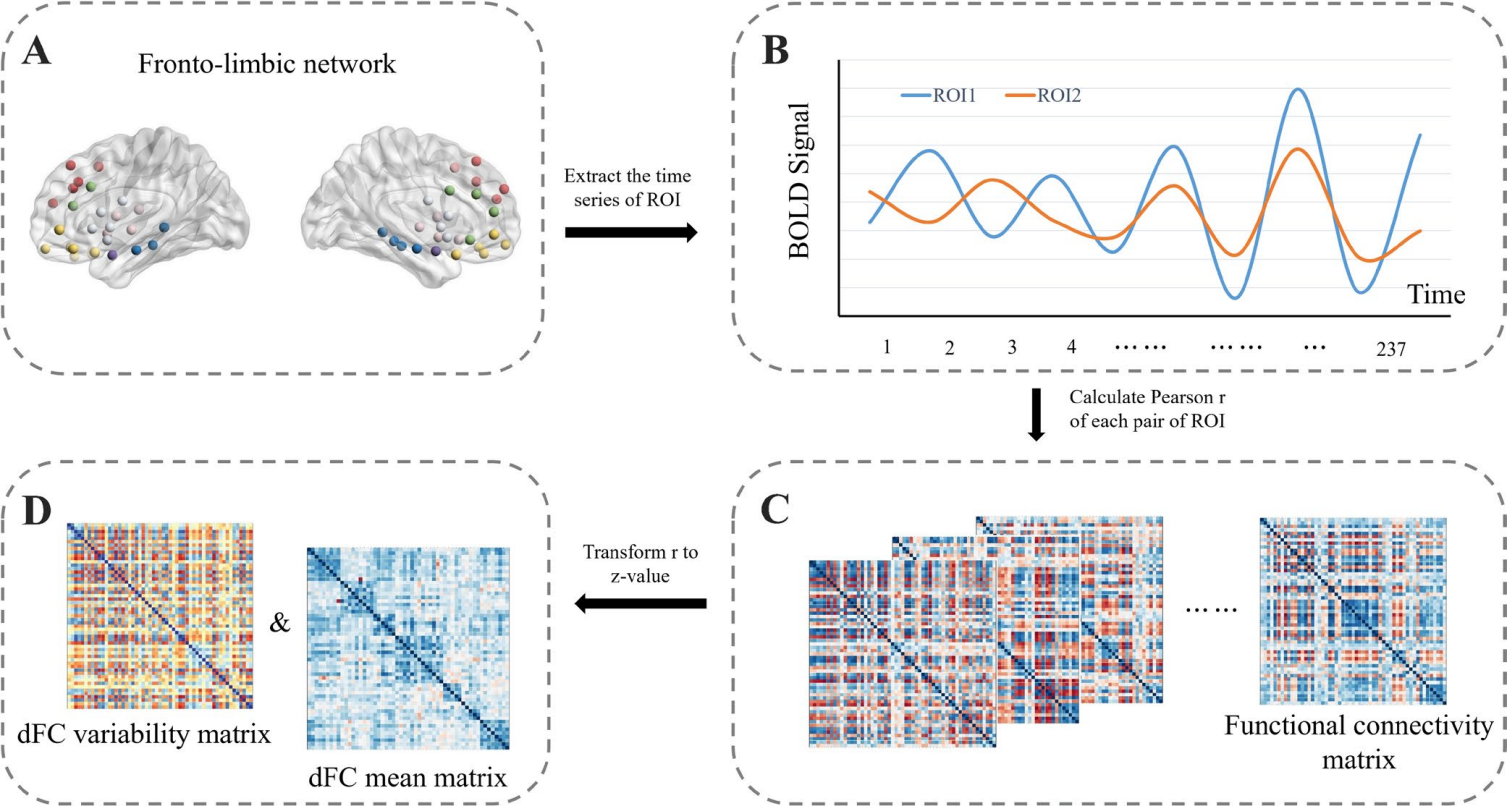
The pipeline of dynamic functional connectivity (dFC) data analysis. (A) Participant BOLD signals were extracted based on the 55 ROIs within the fronto‐limbic network, then (B) ROI‐wise time series were obtained for each participant, and (C) Pearson's correlation coefficients were calculated for each pair of ROIs across 237 time points, resulting in a series of functional connectivity matrices. After that, (D) Fisher‐z transformation was used to derive standard deviation and the mean dFC value.

After calculating pairwise connectivity values, the entire resting fMRI data of each participant was divided into 237 FC matrices, each comprising 3025 connections (55 × 55). Each functional connection matrix was converted into z‐value maps by Fisher's z transformation to improve the normality of the correlation distribution. Then, the mean dFC index, a parameter calculated by the mean value of FC across all the windows derived from the DCC approach, and dFC variability values, representing the standard deviation of 237 functional matrices, were computed and utilized for subsequent analyses. The pipeline of data analysis is depicted in (Figure 1).

In addition, we also analyzed static FC between each ROI within the fronto‐limbic network. Detailed information is provided in Appendix S2.

### 2.4 Statistical Analysis

To evaluate the distribution of demographic data and clinical scores, we performed the Kolmogorov–Smirnov test to assess normality and Levene's test to assess the homogeneity of variances using SPSS 26.0 (SPSS, Chicago, IL, USA). Subsequently, group differences were analyzed using two‐sample *t*‐tests or Mann–Whitney U tests for continuous variables, depending on the results of the normality and variance tests, while $\chi^{2}$ tests were utilized for categorical variables. Third, to investigate the difference in the dFC pattern between the two groups, an independent two‐sample *t*‐test (two‐tailed) was performed on the mean and standard deviation functional matrix of the two groups. After group comparison, mean dFC did not show significant group differences, so the following analysis focused on the dFC variability. After the comparison, variables showing significant group differences were included as covariates in the subsequent correlation and mediation analysis.

The False discovery rate (FDR) correction was applied for multiple comparison correction with q < 0.05. All tests were performed with two‐tailed, and a p value < 0.05 was considered as statistically significant. For the fMRI data analysis, the FDR correction was applied across 1485 unique off‐diagonal functional connections within the 55‐node fronto‐limbic network as the matrices were symmetric.

#### 2.4.1 Classification Analysis

Further, to ascertain the predictive capability of the dFC variability for classifying NSSI and non‐NSSI, a classification analysis was performed. For this analysis, we applied six different machine learning models, including logistic regression with L1 and L2 regularization, Support Vector Machine (SVM) with a Radial Basis Function (RBF) kernel, Random Forest (RF), extreme gradient boosting (XGBoost), and balanced random forest, with DFC variabilities of connections that showed significant group differences served as the classification features. Feature importance was examined within the different classifiers and Model stability was ensured through stratified cross‐validation, repeated train–test splits, varied test size and matched‐group validation analyses. For details of classification analysis see Appendix S1.

To address the imbalance between groups that 154 samples in NSSI group and 50 samples in non‐NSSI group, we applied the Synthetic Minority Oversampling Technique (SMOTE) [35], a method resulting in a balanced dataset for model training. The synthetic data was only used to classification analysis.

#### 2.4.2 Correlation and Mediation Analysis

To investigate the association between dFC variability, severity of depressive symptoms (PHQ‐9 score), and NSSI behavior, category mediation analysis was applied with Mplus [36]. In particular, Pearson correlations were computed between PHQ‐9 scores and values of dFC variability that showed significant group differences. To minimize potential confounding, the correlation analyses adjusted for a range of covariates, including sex and head motion, which differed significantly between groups, as well as age, years of education, family income, anxiety severity (GAD‐7), alcohol use, tobacco use, psychiatric medication status, and family history of psychiatric disorders [37, 38, 39]. Bonferroni correction was applied to account for multiple correlations (*N* = 6).

Then mediation analysis was performed, with depressive symptoms as the independent variable, the dFC variability as the mediator, and the engagement in NSSI behavior as the dependent variable. To make the covariate adjustment consistent with the correlation analyses, the mediation model was adjusted for the same set of covariates. The indirect effect was tested using bias‐corrected bootstrap confidence intervals, with 10,000 bootstrap resamples requested. The total effect, direct effect, and indirect effect were estimated. Additionally, to verify the robustness of the above model, we swapped the positions of the independent and mediating variables and reconstructed an alternative model, in which the dFC variability is the independent variable, the PHQ‐9 score is the mediator, and NSSI behavior is the dependent variable.

### 2.5 Matched‐Group Validation

Due to the number of samples being imbalanced between the two groups, we conducted an additional matched‐group analysis to test the robustness of our results. The matched‐group analysis results were generally consistent. More details and the results can be found in Appendix S3, Tables S4–S6, and Figures S9–S14.

## 3 Results

### 3.1 Demographics and Clinical Characteristics

The analyzed sample comprised 204 adolescents, including 154 MDD patients with NSSI and 50 MDD patients without NSSI. One subject was excluded due to excessive head movement, and 29 subjects were excluded due to incomplete questionnaires. Table 1 shows the demographic and clinical characteristics of 204 participants in this study. The groups differed significantly in gender distribution ($\chi^{2}$= 57.176, p < 0.001) and head motion (t = 2.435, p = 0.016). For clinical assessment, The NSSI group showed higher PHQ‐9 scores than the non‐NSSI group (z = 2.97, *p* = 0.003), indicating more severe depressive symptoms. No significant differences between the two groups were observed in age, education, family income, GAD‐7, alcohol use, tobacco use, or psychiatric medication status.

**TABLE 1:** Demographic and clinical data of participants.

Demographic	NSSI	non‐NSSI	χ2/t/z	*p*
Number of participants	154	50	—	—
Age^a^ (years)	15.12 (1.67)	15.66 (1.61)	−1.741	0.0831
Gender^b^ (male/female)	24/130	24/26	57.176	< 0.001***
Education^a^ (years)	9.57 (1.80)	10.13 (1.66)	−1.662	0.098
Family income^a^ (ten thousand yuan)	7.06 (2.24)	7.16 (1.88)	0.271	0.787
Family history of psychiatry	16	5	—	—
Head motion^a^ (FD)	0.053 (0.027)	0.045 (0.01)	2.435	0.016*
PHQ‐9^c^	20 (15 ~ 24)	16.5 (12 ~ 20)	2.97	0.003**
GAD‐7^c^	13 (9 ~ 17)	12 (7 ~ 15)	1.705	0.08
Alcohol use			1.529	0.465
Never	128	44	—	—
Mild	22	4	—	—
Moderate	4	2	—	—
Tobacco use	—	—	0.334	0.846
Never	141	47	—	—
Smoking in the past	5	1	—	—
Currently smoking	8	2	—	—
Psychiatric medication^b^	7	0	2.353	0.125

Abbreviations: FASM, Functional Assessment of Self‐Mutilation; FD, Framewise displacement; GAD‐7, Seven‐item Generalized Anxiety Disorder Scale; PHQ‐9, Nine‐item Patient Health Questionnaire.

^a^ Independent t‐test.

^b^ $\chi^{2}$ test.

^c^ Mann–Whitney U test.

* p < 0.05.

** p < 0.01.

*** p < 0.001.

### 3.2 Group Difference in dFC Variability Values and Mean Values

**FIGURE 2:**
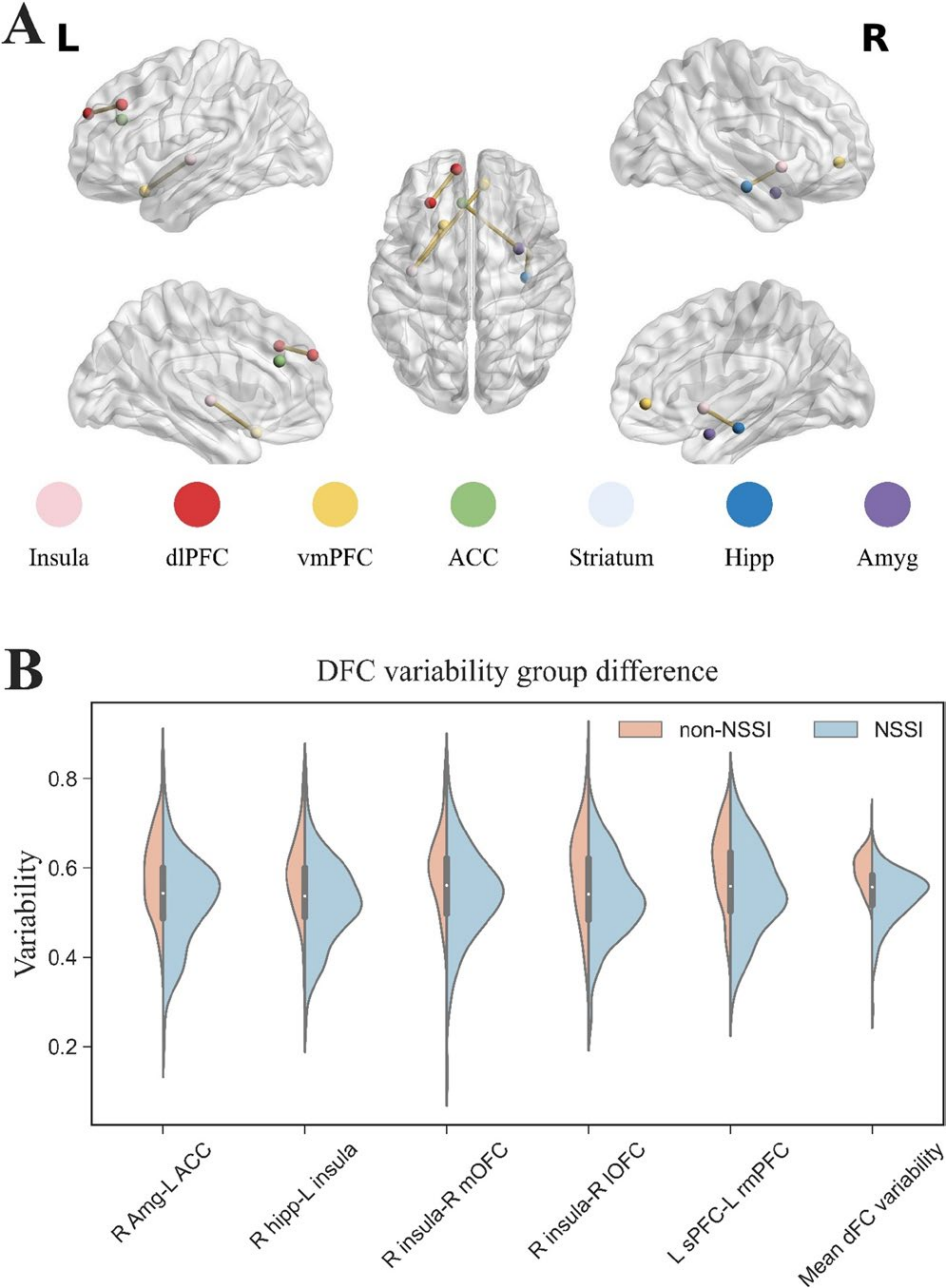
Group differences in dFC variabilities. (A) Connections with significant between‐group differences in dFC variabilities across 55 nodes within the fronto‐limbic network (p < 0.05, FDR correction). Different colors represent different brain regions. (B) Group‐level comparison of dFC variability. dFC, dynamic functional connectivity; NSSI, patients with non‐suicidal self‐injury; non‐NSSI, patients without non‐suicidal self‐injury; FDR, false discovery rate; dlPFC, dorsolateral prefrontal cortex; vmPFC, ventromedial prefrontal cortex; ACC, anterior cingulate cortex. Amg, amygdala; hip, hippocampus; mOFC, medial orbitofrontal cortex; lOFC, lateral orbitofrontal cortex; rmPFC, rostral middle PFC; L, left; R, right.

After comparing the group difference of the dFC variability and applying multiple comparison correction with FDR (q < 0.05), five pairs of connections show significant differences in the dFC variability between the NSSI group and non‐NSSI group, as displayed in (Figure 2 and Table 2). The dFC variability of the NSSI group was lower than that of the non‐NSSI group across all connections. These connections contain 9 nodes in the amygdala, hippocampus, striatum, ACC, vmPFC, and dlPFC. Specifically, the analysis revealed differences in 5 connections: between the right amygdala (x = 31, y = 4, z = −22) and the left ACC (x = −6, y = 34, z = 26; t = −4.251, p < 0.001), between the right hippocampus (x = 36, y = −15, z = −18) and the left insula (x = −38, y = 8, z = −5; t = −4.325, p < 0.001), between the right insula (x = 39, y = −11, z = 0) and the right medial orbitofrontal cortex (OFC) (x = 8, y = 46, z = −2; t = −4.004, p < 0.001), between the right insula (x = 39, y = −11, z = 0) and the left lateral OFC (x = −18, y = 19, z = −21; t = −3.898, p < 0.001), between the left superior PFC (x = −10, y = 56, z = 30) and the left rostral middle PFC (x = −27, y = 34, z = 36; t = −3.795, p < 0.001). The mean dFC variability of these five connections also showed a difference between the two groups (t = −7.267, p < 0.001). In addition, the supplementary static FC analysis yielded non‐significant results, as shown in Table S3.

**TABLE 2:** Connections showing group differences in dFC variabilities (NSSI < non‐NSSI).

	Connections		t	*p*
	R amygdala	L ACC	−4.251	< 0.001
Coordinates	x = 31, y = 4, z = −22	x = −6, y = 34, z = 26		
	R hippocampus	L insula	−4.325	< 0.001
Coordinates	x = 36, y = −15, z = −18	x = −38, y = 8, z = −5		
	R insula	R medial OFC	−4.004	< 0.001
Coordinates	x = 39, y = −11, z = 0	x = 8, y = 46, z = −2		
	R insula	L lateral OFC	−3.898	< 0.001
Coordinates	x = 39, y = −11, z = 0	x = −18, y = 19, z = −21		
	L superior PFC	L rostral middle PFC	−3.795	< 0.001
Coordinates	x = −10, y = 56, z = 30	x = −27, y = 34, z = 36		
	Mean dFC variability		−7.267	< 0.001

*Note:* The coordinate space is MNI (Montreal Neurological Institute); Coordinates (x, y, z; mm) correspond to the centroid of each ROI in MNI space, based on the 268‐node functional brain atlas.

Abbreviations: ACC, anterior cingulate cortex; dFC, dynamic functional connectivity; L, left; non‐NSSI, patients without non‐suicidal self‐injury; NSSI, patients with non‐suicidal self‐injury; OFC, orbitofrontal cortex; PFC, prefrontal cortex; R, right.

### 3.3 Classification Analysis

Across the six models, Logistic Regression with L1 regularization demonstrated the best discriminative performance, achieving the highest mean AUC (0.843) with relatively low variability across splits (mean AUC SD = 0.050). It also yielded the highest mean PR‐AUC (0.944) and the strongest balanced accuracy (0.749), with balanced sensitivity (0.740) and specificity (0.758), indicating stable performance across both classes. Among tree‐based models, Balanced Random Forest showed competitive performance (mean AUC = 0.814, SD = 0.059; PR‐AUC = 0.931), with relatively higher sensitivity (0.787) but lower specificity (0.654). Logistic Regression (L2), RF and XGBoost demonstrated comparable but slightly lower performance. ROC curves, AUC distributions, confusion matrices, performance heatmaps for the different models are shown in (Figures S3–S7) respectively.

Feature importance analyses showed that mean dFC variability was the most influential predictor across models, dominating the L1 logistic regression coefficients, while Balanced Random Forest assigned more evenly distributed importance across features, suggesting a combined contribution of multiple dFC alterations. More details and additional machine‐learning results are provided in Appendix S1 The feature importance results are shown in (Figure S6).

### 3.4 Correlation Analysis and Mediation Analysis

Correlation analysis revealed that dFC variabilities, particularly the mean dFC variability were inversely correlated with PHQ‐9 scores (r = −0.228, p = 0.001). After Bonferroni correction, only the mean dFC variability survives. Other correlation results could be found in Table S2 in Supporting Information and scatterplot found in (Figure S8).

**FIGURE 3:**
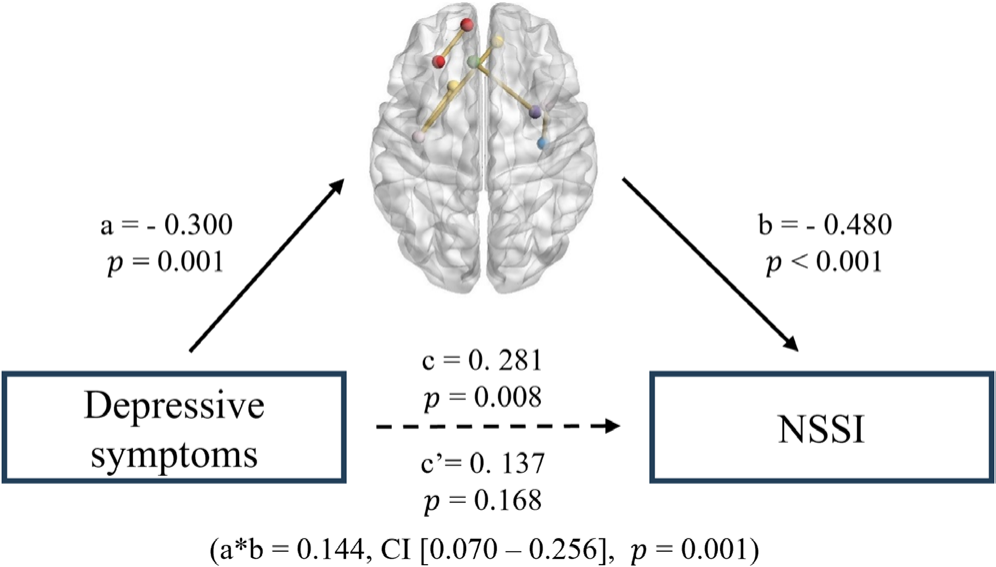
Mediation model illustrating the relationship between severity of depressive symptoms, NSSI (non‐suicidal self‐injury) behavior, and the mean dFC variability. The analysis reveals a significant mediating role of the mean dFC variability in the association between depression severity and NSSI engagement. Covariates used in the models included sex, head motion, age, years of education, family income, anxiety severity (GAD‐7), alcohol use, tobacco use, psychiatric medication status, and family history of psychiatric disorders.

Mediation analysis showed that mean dFC variability significantly mediated the association between depressive symptom severity and NSSI engagement. The indirect effect was significant (a*b = 0.144, 95% bootstrap CI [0.070, 0.256], p = 0.001; Figure 3). The total effect of depressive symptoms on NSSI was c = 0.281 (SE = 0.105, p = 0.008), and the direct effect was not significant after accounting for the mediator (c = 0.137, SE = 0.100, p = 0.168).

In addition, we tested an alternative mediation model in which mean dFC variability was specified as the independent variable, PHQ‐9 score as the mediator, and NSSI behavior as the outcome. The indirect effect was significant (a*b = −0.038, 95% bootstrap CI [−0.086, −0.011], *p* = 0.039) and substantially smaller than that observed in the hypothesized mediation model.

## 4 Discussion

The current study investigated functional dynamics within the fronto‐limbic network to understand the NSSI behaviors in depressive adolescents. Our findings revealed that depressed adolescents with NSSI displayed reduced dFC variability, which mediated the relationship between depressive severity and NSSI propensity. Altered connectivity patterns within key brain regions including the amygdala, ACC, hippocampus, insula, and OFC, showed good discriminative performance, with a mean AUC of 0.843. To our knowledge, this study represents the first application of the dFC method in NSSI research, further delving into the relationship between depressive symptoms and NSSI behaviors on the neural level. These findings suggest that temporal dynamic patterns of the fronto‐limbic network serve as candidate neural features for NSSI, and may highlight a novel neural target for NSSI interventions.

### 4.1 The Predictive Power of dFC Variability in NSSI

Our study utilizes dFC analysis to offer a more sensitive reflection of the brain's inherently fluctuating connectivity compared to static methods [14, 15]. In our sample, static FC analysis fell short in distinguishing MDD youth with and without NSSI. By contrast, dFC variability identified group differences, suggesting that temporally dynamic features may be informative for NSSI‐related heterogeneity within MDD.

The observed reduction in dFC variability among adolescents with depression and NSSI suggests a less flexible, and potentially maladaptive, pattern of neural communication within the fronto‐limbic network. Reduced dFC variability reflects diminished temporal fluctuation in inter‐regional interactions, indicating lower neural flexibility and a limited capacity for adaptive network reconfiguration in response to emotional or contextual demands. Similar alterations in dFC variability have been consistently reported in MDD [40, 41, 42], reflecting abnormally synchronized and rigid neural dynamics associated with rumination, slowed information processing, and reduced cognitive flexibility [43, 44]. Such rigidity may underlie difficulties in shifting emotional states and maladaptive coping behaviors such as NSSI [45, 46]. Supporting this interpretation, adolescents with NSSI have shown impaired executive functioning and cognitive flexibility [47], suggesting that reduced dFC variability could serve as a neural correlate of cognitive rigidity.

Alternatively, decreased dFC variability may reflect reduced information transfer or less efficient communication between brain regions within the fronto‐limbic circuit. Previous studies have demonstrated that dynamic connectivity is closely related to communication efficiency and information exchange across networks [44, 48]. Within this framework, diminished dFC variability could indicate weakened integration of emotional and regulatory processes, leading to impaired adaptability under stress. Moreover, this phenomenon may correspond to reduced metastability—the brain's ability to flexibly switch between transient network states. Lower metastability has been linked to cognitive rigidity and persistent negative thinking in depression [49], suggesting that adolescents with depression and NSSI may exhibit a similar reduction in dynamic neural flexibility. Together, these interpretations converge on the idea that decreased dFC variability represents less efficient and less adaptable fronto‐limbic communication, potentially underpinning both cognitive rigidity and maladaptive emotion regulation strategies in NSSI [43, 47].

### 4.2 The Mediating Role of the dFC Variability Between Depressive Symptoms and NSSI

The hypothesized mediation model offers additional insight, suggesting that dFC variability may represent a potential pathway linking depressive severity to NSSI. This suggests that the temporal dynamics of neural connectivity significantly contribute to the pathway from depressive symptoms to NSSI behaviors.

The findings provide neural substrates for the hypothesis that impaired emotional regulation, characteristic of MDD, may predispose individuals to resort to NSSI as a maladaptive coping strategy. First, depressive symptoms are associated with decreased dFC variabilities among MDD, which is consistent with previous studies. Previous studies showed decreased strength or fluctuation in dFC of amygdala subregions [50], the limbic network, the DMN [42], and the frontoparietal network [41], suggesting that aberrant dFC patterns may be large‐scale in regions responsible for emotional regulation and executive function. Depressive states often involve impaired emotional regulation and heightened negative affect, which could disrupt the normal dynamic interplay between regions such as the prefrontal cortex and the limbic system. These disruptions may manifest as reduced dFC variability, indicating less information processing and communication and a more rigid neural response pattern. Such patterns, in turn, limit the brain's ability to effectively modulate emotional responses, exacerbating depressive symptoms. What's worse, earlier maturation of subcortical regions than prefrontal systems in the developing brain of adolescents leads to a lack of top‐down regulation. Consequently, adolescents with depression resort to NSSI as an emotional regulation skill to relieve emotional pain [16, 51, 52]. Furthermore, the fronto‐limbic network's involvement in reward processing suggests that disruptions in its dFC could contribute to the anhedonia commonly observed in MDD. Meanwhile, it may mistakenly connect NSSI with a reward, making NSSI habitual. Consistently, the neurodevelopmental model proposed by Auerbach et al. (2021) [14] suggests that mental disorders as distal factors do harm to specific neural circuitry, such as the fronto‐limbic network, resulting in ideation and behavior of NSSI. Therefore, our findings underscore the importance of dynamic neural interactions within this network in the pathophysiology of depressive symptoms to NSSI.

While it could be a possible pathway leading to NSSI in depressed adolescents, it is important to be noted that our findings based on the cross‐sectional data, and therefore the mediation model reflects associations rather than causal relationship. Future longitudinal studies are needed to clarify the directionality of these effects.

### 4.3 Key Regions of the Fronto‐Limbic Network Mediating Depressive Symptoms to NSSI

In this study, anterior insula showed aberrant dFC with regions, such as hippocampus and parts of OFC in NSSI patients. These results were aligned with previous structural MRI studies linking insula gray matter volume to NSSI behavior [53, 54, 55, 56]. In addition, altered insula activity in NSSI individuals has been observed during processing unpleasant haptic sensations [34], socio‐affective pain [57], or discriminate unpleasant stimuli, reflecting increased physiological arousal to negative stimuli. As a salience‐network hub involved in pain, insular alteration may reflect lower pain sensitivity and altered pain perception in adolescent NSSI [58]. A novel aspect of our findings is the highlighted role of the insula‐hippocampus functional connection network, which to our knowledge, this connection is less frequently examined in the NSSI literature. The hippocampus is integral to emotional regulation and memory processing [16, 59, 60], and involved in the automatic retrieval of emotional and NSSI‐related material [16]. Our results showed young patients with NSSI exhibited abnormal connectivity between the hippocampus and insula, indicating atypical affective pain processing pathway might deteriorate due to negative emotional memories.

The prefrontal system, especially the OFC exhibited abnormal connection with the insula. It is well known that the OFC is parceled into two subregions [61, 62], with the lateral OFC responsible for punishment and subjective valuation of reward and the medial OFC responsible for value‐guided decision making and inhibiting impulsive behaviors [61, 63]. Therefore, for the result of our research, altered dFC between the insula and both the medial OFC and lateral OFC in NSSI patients may contribute to NSSI in distinct ways. Insula‐ medial OFC connectivity may lead to difficulties in evaluating the emotional significance of potential rewards and punishments. While insula‐lOFC connectivity predominantly impairs the ability to accurately and safely assess risk and make decisions.

Indeed, prior neuroimaging studies using static FC analyses in depression have consistently reported fronto‐limbic dysconnectivity, particularly involving the amygdala, ACC, insula, and OFC [22, 64, 65, 66], supporting the central role of disrupted emotion regulation networks in depression. Our findings extend these results by demonstrating that such abnormalities also exist at the dynamic level of neural interactions. Specifically, adolescents with NSSI exhibited reduced dFC variability within the fronto‐limbic network, suggesting a less flexible and more rigid neural communication pattern. This temporal rigidity may reflect more severe impairment in adaptive modulation of emotional and cognitive processes, distinguishing NSSI depression from typical MDD. Therefore, while previous studies identified static alterations in FC across depressive populations, our results highlight that the temporal dynamics of these connections may serve as candidate neural features for NSSI within MDD.

### 4.4 Limitations

There are some limitations in this study. First, owing to the cross‐sectional design, no causal inferences could be drawn regarding the relationship between aberrant dFC and NSSI. Longitudinal studies are needed to clarify the temporal relationship between depressive symptoms, NSSI, and their neural correlates. Second, although our clinical control design can isolate NSSI‐related neural alterations within a depressed population, the absence of a healthy control (HC) group is a notable limitation. While our approach differentiates NSSI‐specific patterns from general MDD features, the lack of an HC group precludes comparisons with typical neurodevelopmental trajectories. Consequently, we cannot determine how these dFC alterations deviate from healthy brain function or if they represent a unique interaction between depressive pathology and self‐injury. Future research utilizing a three‐group design, which incorporates healthy controls alongside MDD patients with and without NSSI, is warranted to fully disentangle these complex neurobiological effects. Third, given established sex differences in the prevalence and clinical presentation of adolescent NSSI, as well as evidence for sex‐related brain structural differences in MDD, part of the between‐group effects may reflect sex‐related heterogeneity rather than NSSI status alone, although sex was included as a covariate in the analyses [20, 67]. Finally, the neurobiology analysis exclusively focuses on dFC within the fronto‐limbic network. However, NSSI may also involve alterations in other large‐scale brain networks, such as the default mode network and salience network, and their interactions. Thus, future studies examining how across‐network dynamics are related to NSSI would be required to provide a more comprehensive understanding of its neural basis.

## 5 Conclusion

In summary, our findings suggest that the altered variation of FC within the fronto‐limbic network especially connections between the insula, ACC, OFC, and hippocampus, may underlie NSSI among adolescents with MDD. These abnormal links mediate the relationship between depressive symptoms and NSSI, highlighting the front‐limbic network as a new target for therapeutic interventions. Furthermore, these results suggest that depressed adolescents with NSSI show distinct neural patterns in emotion processing, risk assessment, decision‐making, and pain perception compared to those without NSSI. These findings suggest a distinct neural profile associated with NSSI status within adolescents with MDD. This underscores the importance of considering NSSI‐associated neural alterations within the MDD population, rather than merely a symptom of depression severity. Moreover, our preliminary machine learning analysis suggests that fronto‐limbic dFC variability holds potential as a candidate neural feature for identifying NSSI within the MDD population. We advocate for greater utilization of dFC analysis in future research for its deeper insights into NSSI's neural basis.

## Author Contributions

Shengmiao Ni, Yongjie Zhou, Junrong Han: Conceptualization, Formal analysis, Visualization, Methodology, Writing original draft; Wei Zhang, Binke Yuan, Xinni Luo, Haiyan Li: Data curation, Formal analysis, Visualization, Software; Nan Yan, Lan Wang, Lingyun Zeng: Validation, Resources, Writing review and editing; Xuchu Weng, Lijuan Huo: Conceptualization, Resources, Funding acquisition, Writing review and editing, Supervision.

## Funding

This work was supported by the National Natural Science Foundation of China, 82102673, U23B2018, 62271477. Natural Science Foundation of Guangdong Province, 2023A1515011802, 2024A1515010527; 2023A1515010840. Philosophy and Social Science Project of Guangdong Province, GD22YXL03. Striving for the First‐Class, Improving Weak Links and Highlighting Features (SIH) Key Discipline for Psychology in South China Normal University.

## Ethics Statement

This study was approved by the Research Ethics Committee of Shenzhen Kangning Hospital (IRB: 2020‐K021–02) and all participants and their guardians have signed informed consent.

## Conflicts of Interest

The authors declare no conflicts of interest.

## Supporting information


**Table S1:** Coordinates of 55 nodes of the fronto‐limbic circuitry. ACC, anterior cingulate cortex; dPFC, dorsal prefrontal cortex; vPFC, ventral prefrontal cortex.
**Table S2:** Means, standard deviations, and bivariate correlations for all study variables (*n* = 204).
**Table S3:** Top 5 static FC connections with uncorrected *p*‐value.
**Table S4:** Demographic and clinical characteristics of participants in matched group.
**Table S5:** Connections showing group differences in dFC variability in matched group (NSSI < non—NSSI).
**Table S6:** Means, standard deviations, and bivariate correlations for all study variables in matched group.
**Figure S1:** Flow chart for study participants. The flow chart shows the progression of participants throughout this study.
**Figure S2:** Illustration of the 55 nodes of the fronto‐limbic network. Nodes are color‐coded to represent different parcellations. dlPFC, dorsolateral prefrontal cortex; vmPFC, ventromedial prefrontal cortex; ACC, anterior cingulate cortex.
**Figure S3:** ROC curves by different models with 50 splits. Six different ROC curves of classification models in distinguishing between NSSI and non‐NSSI. The logistic regression model with L1 has the best model performance with mean AUC of 0.843, while support vector model with RBF kernel showed the worst with mean AUC of 0.432.
**Figure S4:** Box plot of AUC distribution across models. distribution of test‐set ROC‐AUC values across all repeated stratified splits and test sizes for each classifier (LR‐L1, LR‐L2, SVM‐RBF, Random Forest, XGBoost, and Balanced Random Forest). The dashed horizontal line marks chance‐level performance (AUC = 0.50). Overall, LR with L1 regularization demonstrated the highest and most stable AUC distribution, whereas SVM‐RBF showed marked variability and lower performance.
**Figure S5:** Confusion matrices averaged across splits. Top row: Mean confusion matrices across repeated stratified splits for each model, computed on the untouched test sets using a classification threshold determined from training‐set out‐of‐fold predictions (Youden index). Bottom row: Corresponding normalized confusion matrices, with diagonal entries reflecting specificity and sensitivity. Class 1 indicates NSSI and Class 0 indicates non‐NSSI.
**Figure S6:** Feature importance by model across splits. Feature importance rankings are summarized across all repeated runs for models that provide interpretable importance estimates. For Logistic Regression (L1/L2), importance is defined as the absolute value of standardized model coefficients; for tree‐based models (Random Forest, XGBoost, Balanced Random Forest), importance is derived from model‐specific impurity/gain‐based measures. Bars show mean importance and error bars indicate standard deviation across splits. V1–V6 denote the selected dynamic functional connectivity (dFC) features (connections) used for classification. Results indicate that mean dFC variability consistently contributed strongly to prediction, while ensemble models distributed importance more evenly across features.
**Figure S7:** Heatmap of performance metrics across models and test sizes. Heatmaps summarize mean classification metrics for each model as a function of hold‐out test size (48, 56, 64, 72, and 80 subjects), averaged across repeated stratified splits. Metrics include ROC‐AUC, sensitivity, specificity, F1‐score, Matthews correlation coefficient (MCC), and balanced accuracy. Warmer colors indicate better performance.
**Figure S8:** Partial correlation between depressive symptoms (PHQ‐9 score) and brain dynamic functional connectivity variability. The mean dynamic functional connectivity variability across all group‐differentiated connections was significantly correlated with PHQ‐9 score (Pearson r = −0.228, *p* = 0.001) after controlling covariates of age, sex, years of education, family income, GAD‐7 score, alcohol use, tobacco use, psychiatric medication, family history of psychiatry and head motion.
**Figure S9:** ROC curves by different models in matched group. Five different ROC curves of classification models in distinguishing between NSSI and non‐NSSI. The logistic regression model with L2 has the best model performance with mean AUC of 0.81, while support vector model with RBF kernel showed the worst with mean AUC of 0.505.
**Figure S10:** Box plot of AUC distribution across models and test size in matched group. distribution of test‐set ROC‐AUC values across all repeated stratified splits and test sizes for each classifier (LR‐L1, LR‐L2, SVM‐RBF, Random Forest, XGBoost, and Balanced Random Forest). The dashed horizontal line marks chance‐level performance (AUC = 0.50). Overall, LR with L2 regularization demonstrated the highest and most stable AUC distribution, whereas SVM‐RBF showed marked variability and lower performance.
**Figure S11:** Confusion matrices averaged across splits in matched group. Top row: Mean confusion matrices across different splits ratio for each model, computed on the test sets using a classification threshold determined from training‐set out‐of‐fold predictions (Youden index). Bottom row: Corresponding normalized confusion matrices, with diagonal entries reflecting specificity and sensitivity. Class 1 indicates NSSI and Class 0 indicates non‐NSSI.
**Figure S12:** Feature importance by model across splits in matched group. Feature importance rankings are summarized across all different split ratio runs for models that provide interpretable importance estimates. For Logistic Regression (L1/L2), importance is defined as the absolute value of standardized model coefficients; for tree‐based models (Random Forest, XGBoost, Balanced Random Forest), importance is derived from model‐specific impurity/gain‐based measures. Bars show mean importance and error bars indicate standard deviation across splits. V1–V6 denote the selected dynamic functional connectivity (dFC) features used for classification.
**Figure S13:** Heatmap of performance metrics across models in matched group. Heatmaps summarize mean classification metrics for each model across different split ratio (training size/test size, 80/20, 70/30, 60/40). Metrics include ROC‐AUC, sensitivity, specificity, F1‐score, Matthews correlation coefficient (MCC), and balanced accuracy. Warmer colors indicate better performance.
**Figure S14:** Mediation model of severity of depression, NSSI behavior and mean dFC variability in matched group. The significant result of mediation analysis showed that the dynamic functional connectivity (dFC) variability links to the severity of depression and NSSI behavior. Path a*b represents an indirect path which is the relationship between depression and NSSI behavior that is mediated by the mean variability of dynamic FC links which were significantly correlated with the depression.
**Appendix S1:** Methods and Results.
**Appendix S2:** Static functional connectivity (FC) analysis.
**Appendix S3:** Matched group analysis.

## Data Availability

The data that support the findings of this study are available on request from the corresponding author. The data are not publicly available due to privacy or ethical restrictions.
