# Supplementary material for: Abnormal Dynamic Functional Connectivity Within the Fronto‐Limbic Network Mediates the Association Between Depressive Symptoms and Non‐Suicidal Self‐Injury in Adolescents
Source: CNS Neurosci Ther. 2026 Jul 21;32(7):e71023. doi: 10.1002/cns.71023 (PMC13389438; doi:10.1002/cns.71023)
Supplement: Supplementary file 1 — Table S1: Coordinates of 55 nodes of the fronto‐limbic circuitry. ACC, anterior cingulate cortex; dPFC, dorsal prefrontal cortex; vPFC, ventral prefrontal cortex. Table S2: Means, standard deviations, and bivariate correlations for all study variables (n = 204). Table S3: Top 5 static FC connections with uncorrected p‐value. Table S4: Demographic and clinical characteristics of participants in matched group. Table S5: Connections showing group differences in dFC variability in matched group (NSSI < non—NSSI). Table S6: Means, standard deviations, and bivariate correlations for all study variables in matched group. Figure S1: Flow chart for study participants. The flow chart shows the progression of participants throughout this study. Figure S2: Illustration of the 55 nodes of the fronto‐limbic network. Nodes are color‐coded to represent different parcellations. dlPFC, dorsolateral prefrontal cortex; vmPFC, ventromedial prefrontal cortex; ACC, anterior cingulate cortex. Figure S3: ROC curves by different models with 50 splits. Six different ROC curves of classification models in distinguishing between NSSI and non‐NSSI. The logistic regression model with L1 has the best model performance with mean AUC of 0.843, while support vector model with RBF kernel showed the worst with mean AUC of 0.432. Figure S4: Box plot of AUC distribution across models. distribution of test‐set ROC‐AUC values across all repeated stratified splits and test sizes for each classifier (LR‐L1, LR‐L2, SVM‐RBF, Random Forest, XGBoost, and Balanced Random Forest). The dashed horizontal line marks chance‐level performance (AUC = 0.50). Overall, LR with L1 regularization demonstrated the highest and most stable AUC distribution, whereas SVM‐RBF showed marked variability and lower performance. Figure S5: Confusion matrices averaged across splits. Top row: Mean confusion matrices across repeated stratified splits for each model, computed on the untouched test sets using a classification threshold dete [file CNS-32-e71023-s001.docx]

**Supplementary Information:**

**Abnormal dynamic functional connectivity within the fronto-limbic network mediates the association between depressive symptoms and non-suicidal self-injury in adolescents**

**Contents**

[Supplementary Tables 1](#_Toc223195736)

[Supplementary Figures 8](#_Toc223195737)

[Appendix S1: Methods and Results 22](#_Toc223195738)

[Clinical assessment 22](#_Toc223195739)

[Functional Assessment of Self-Mutilation (FASM) 22](#_Toc223195740)

[Patient Health Questionnaire-9 (PHQ-9) 22](#_Toc223195741)

[Generalized Anxiety Disorder Scale 7-item (GAD-7) 22](#_Toc223195742)

[MRI data acquisition 23](#_Toc223195743)

[fMRI data pre-processing 23](#_Toc223195744)

[Classification analysis 24](#_Toc223195745)

[Methods 24](#_Toc223195746)

[Results 26](#_Toc223195747)

[Appendix S2: Static functional connectivity (FC) analysis 29](#_Toc223195748)

[Static functional connectivity (FC) analysis between two groups 29](#_Toc223195749)

[Appendix S3: Matched group analysis 30](#_Toc223195750)

[Matched group analysis 30](#_Toc223195751)

[Demographics and clinical characteristics 30](#_Toc223195752)

[Group difference in dFC variability values 31](#_Toc223195753)

[Classification analysis 31](#_Toc223195754)

[Correlation analysis and mediation analysis 33](#_Toc223195755)

[Reference 34](#_Toc223195756)

Supplementary Tables

**Table S1.** Coordinates of 55 nodes of the fronto-limbic circuitry. ACC, anterior cingulate cortex; dPFC, dorsal prefrontal cortex; vPFC, ventral prefrontal cortex.

=

| **Table S2**. Means, standard deviations, and bivariate correlations for all study variables (n=204) | | | | | | | | |
| --- | --- | --- | --- | --- | --- | --- | --- | --- |
| **Correlation** | ***M(SD)*** | **1** | **2** | **3** | **4** | **5** | **6** | **7** |
| 1.PHQ-9 | 18.20 ± 6.06 | 1 |  |  |  |  |  |  |
| 2. R amygdala-L ACC | 0.539 ± 0.100 | -0.129 |  |  |  |  |  |  |
| 3. R hippocampus-L insula | 0.538 ± 0.093 | -0.139 | 0.141 |  |  |  |  |  |
| 4. R insula-R medial OFC | 0.555 ± 0.099 | -0.113 | 0.208^**^ | 0.218^**^ |  |  |  |  |
| 5. R insula-L lateral OFC | 0.548 ± 0.097 | -0.132 | 0.114 | 0.169^*^ | 0.292^***^ |  |  |  |
| 6. L superior PFC-L rostral middle PFC | 0.564 ± 0.097 | -0.147^*^ | 0.196^**^ | 0.073 | 0.116 | 0.134 |  |  |
| 7. Mean dFC variability | 0.547 ± 0.055 | -0.228^***^ | 0.586^***^ | 0.537^***^ | 0.639^***^ | 0.596^***^ | 0.526^***^ |  |
| Covariates: age, sex, years of education, family income, GAD-7 score, alcohol use, tobacco use, psychiatric medication, family history of psychiatry and head motion. | | | | | | | | |
| Abbreviation: ACC, anterior cingulate cortex; dFC, dynamic functional connectivity; PFC, prefrontal cortex; OFC, orbitofrontal cortex; PHQ-9, the Patient Health Questionnaire-9; R, right; L, left | | | | | | | | |
| *, p <0.05; **, p< 0.01; ***, p<0.001 | | | | | | | | |

| **Table S3.** Top 5 static FC connections with uncorrected p-value. | | | | | | |
| --- | --- | --- | --- | --- | --- | --- |
| Rank | Connections | | t | p-value | q(FDR) | |
| 1 | R-Insula | R-Hippocampus | 4.238 | < 0.001 | 0.070 | |
| Coordinates | x = 39, y = -11, z = 0 | x = 29, y = -37, z = 0 |  |  |  |  |
| 2 | R-Hippocampus | R-Hippocampus | 3.292 | 0.001 | 0.563 | |
| Coordinates | x = 36, y = -15, z = -18 | x = 29, y = -37, z = 0 |  |  |  |  |
| 3 | L-Para Hippocampal | R-Hippocampus | 2.972 | 0.004 | 0.563 | |
| Coordinates | x = -32, y = -40, z = -4 | x = 29, y = -37, z = 0 |  |  |  |  |
| 4 | L-Rectus | R-Hippocampus | -2.960 | 0.004 | 0.563 | |
| Coordinates | x = -8, y = -40, z = -21 | x = 29, y = -37, z = 0 |  |  |  |  |
| 5 | R-Para Hippocampal | R-Hippocampus | 2.762 | 0.007 | 0.563 | |
| Coordinates | x = 28, y = -28, z = -14 | x = 36, y = -15, z = -18 |  |  |  |  |
| **Note.** Coordinates are in Montreal Neurological Institute (MNI) space (mm) and correspond to ROI centroids in the 268-node functional atlas. L = left; R = right; ACC = anterior cingulate cortex; dFC = dynamic functional connectivity; OFC = orbitofrontal cortex; PFC = prefrontal cortex; R/L = right/left; NSSI = non-suicidal self-injury. | | | | | | |

| **Table S4**. Demographic and clinical characteristics of participants **in matched group** | | | | |  |
| --- | --- | --- | --- | --- | --- |
| **Characteristic** | **NSSI (n=49)** | **Non-NSSI (n=49)** | **Statistic** | **P value** |  |
| Age, years | 15.45 (1.51) | 15.61 (1.59) | t = -0.520 | 0.604 |  |
| Sex, No. (%) |  |  |  |  |  |
| Male | 23 (46.9) | 23 (46.9) |  |  |  |
| Female | 26 (53.1) | 26 (53.1) |  |  |  |
| Education, years | 9.71 (1.53) | 10.09 (1.66) | t = -1.140 | 0.257 |  |
| Family income, 10,000 CNY | 7.67 (1.77) | 7.16 (1.88) | t = 1.376 | 0.172 |  |
| Family history of psychiatric disorders, No. (%) | 3 (6.1) | 5 (10.0) |  |  |  |
|  |  |  |  |  |  |
| Head motion (mean FD) | 0.064 (0.03) | 0.045 (0.01) | t = 3.372 | 0.001 |  |
| PHQ-9 | 18 (15–23) | 16 (12–20) | z = 2.096 | 0.036 |  |
| GAD-7 | 13 (9–17) | 12 (7–15) | z = 1.071 | 0.284 |  |
| Alcohol use, No. (%) |  |  | χ² = 2.689 | 0.101 |  |
| Never | 38 (77.6) | 44 (89.8) |  |  |  |
| Mild | 9 (18.4) | 3 (6.1) |  |  |  |
| Moderate | 2 (4.1) | 2 (4.1) |  |  |  |
| Tobacco use, No. (%) |  |  | χ² = 1.071 | 0.301 |  |
| Never | 42 (85.7) | 46 (93.9) |  |  |  |
| Smoking in the past | 2 (4.1) | 1 (2.0) |  |  |  |
| Currently smoking | 5 (10.2) | 2 (4.1) |  |  |  |
| Psychiatric medication, No. (%) | 5 (10.2) | 0 (0.0) | χ² = 2.353 | 0.125 |  |
| **Note.** Values are mean (SD) unless otherwise indicated. PHQ-9 and GAD-7 are presented as median (range). FD = framewise displacement; PHQ-9 = Patient Health Questionnaire-9; GAD-7 = Generalized Anxiety Disorder-7. P values are from independent-samples t tests for normally distributed continuous variables, Mann–Whitney U tests for non-normally distributed variables, and χ² tests for categorical variables. | | | | |  |

| **Table S5.** Connections showing group differences in dFC variability **in matched group** (NSSI < non - NSSI) | | | | |
| --- | --- | --- | --- | --- |
| **Connection** | **ROI 1 MNI  (x, y, z)** | **ROI 2 MNI  (x, y, z)** | **t** | **P value** |
| R amygdala – L ACC | 31, 4, -22 | -6, 34, 26 | -2.373 | 0.02 |
| R hippocampus – L insula | 36, -15, -18 | -38, 8, -5 | -3.657 | < .001 |
| R insula – R medial OFC | 39, -11, 0 | 8, 46, -2 | -4.447 | < .001 |
| R insula – L lateral OFC | 39, -11, 0 | -18, 19, -21 | -2.955 | 0.004 |
| L superior PFC – L rostral middle PFC | -10, 56, 30 | -27, 34, 36 | -3.620 | < .001 |
| Mean dFC variability | — | — | -5.778 | < .001 |
| **Note.** Coordinates are in Montreal Neurological Institute (MNI) space (mm) and correspond to ROI centroids in the 268-node functional atlas. L = left; R = right; ACC = anterior cingulate cortex; dFC = dynamic functional connectivity; OFC = orbitofrontal cortex; PFC = prefrontal cortex; R/L = right/left; NSSI = non-suicidal self-injury. | | | | |

| **Table S6**. Means, standard deviations, and bivariate correlations for all study variables **in matched group** | | | | | | | | |
| --- | --- | --- | --- | --- | --- | --- | --- | --- |
| **correlation** | ***M(SD)*** | **1** | **2** | **3** | **4** | **5** | **6** | **7** |
| 1.PHQ-9 | 17.13±6.39 | 1 |  |  |  |  |  |  |
| 2. Right amygdala-L ACC | 0.566±0.100 | -0.033 |  |  |  |  |  |  |
| 3. R hippocampus-L insula | 0.553±0.098 | -0.103 | 0.058 |  |  |  |  |  |
| 4. R insula-R medial OFC | 0.556±0.100 | -0.189 | 0.276^**^ | 0.255^*^ |  |  |  |  |
| 5. R insula-L lateral OFC | 0.562±0.100 | -0.183 | 0.263^*^ | 0.208^*^ | 0.309^**^ |  |  |  |
| 6. L superior PFC-L rostral middle PFC | 0.578±0.091 | -0.224^*^ | 0.229^*^ | 0.135 | 0.175 | 0.243^*^ |  |  |
| 7. Mean dFC variability | 0.563±0.063 | -0.236^*^ | 0.617^***^ | 0.532^***^ | 0.657^***^ | 0.669^***^ | 0.574^***^ |  |
| **Note.** Confounding variables: age, sex, years of education, family income, anxiety severity (GAD-7 score), alcohol use, tobacco use, psychiatric medication status, family history of psychiatric disorders, and head motion. | | | | | | | | |
| Abbreviation: ACC, anterior cingulate cortex; dFC, dynamic functional connectivity; OFC, orbitofrontal cortex; PHQ, Patient Health Questionnaire; R, right; L, left | | | | | | | | |
| *, p <0.05; **, p< 0.01; ***, p<0.001 | |  |  |  |  |  |  |  |

Supplementary Figures


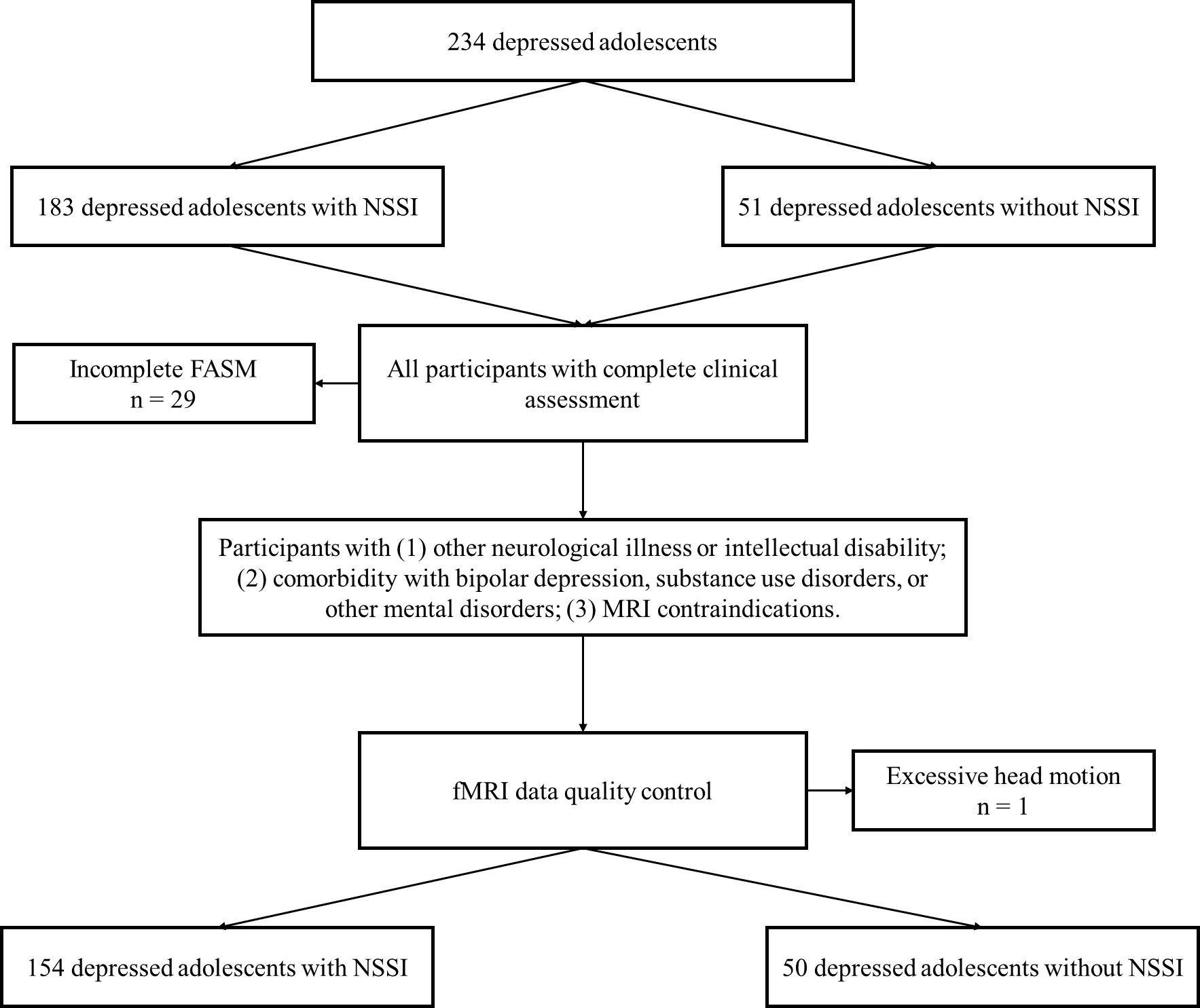


**Figure S1** Flow chart for study participants. The flow chart shows the progression of participants throughout this study.

FASM, Functional Assessment of Self-Mutilation; NSSI, non-suicidal self-injury.


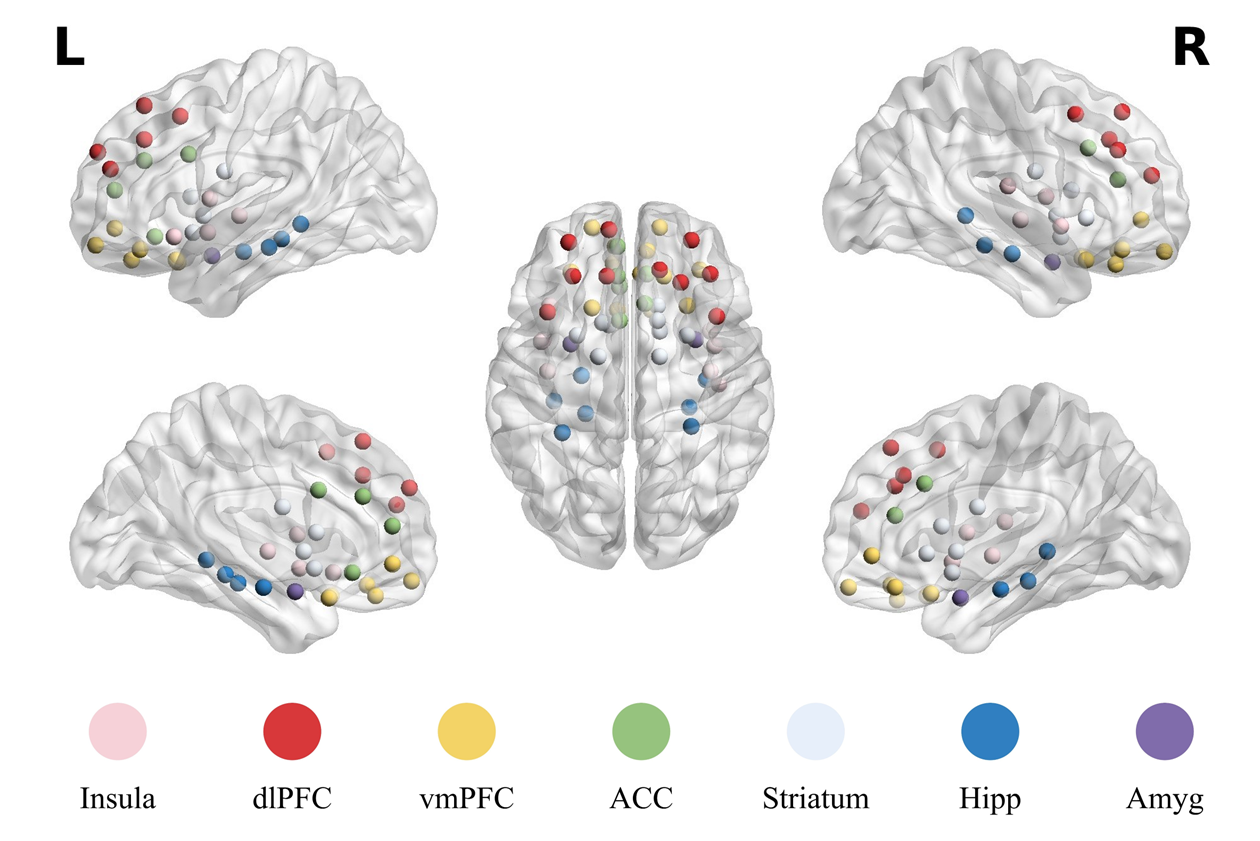


**Figure S2.** Illustration of the 55 nodes of the fronto-limbic network. Nodes are color-coded to represent different parcellations. dlPFC, dorsolateral prefrontal cortex; vmPFC, ventromedial prefrontal cortex; ACC, anterior cingulate cortex.


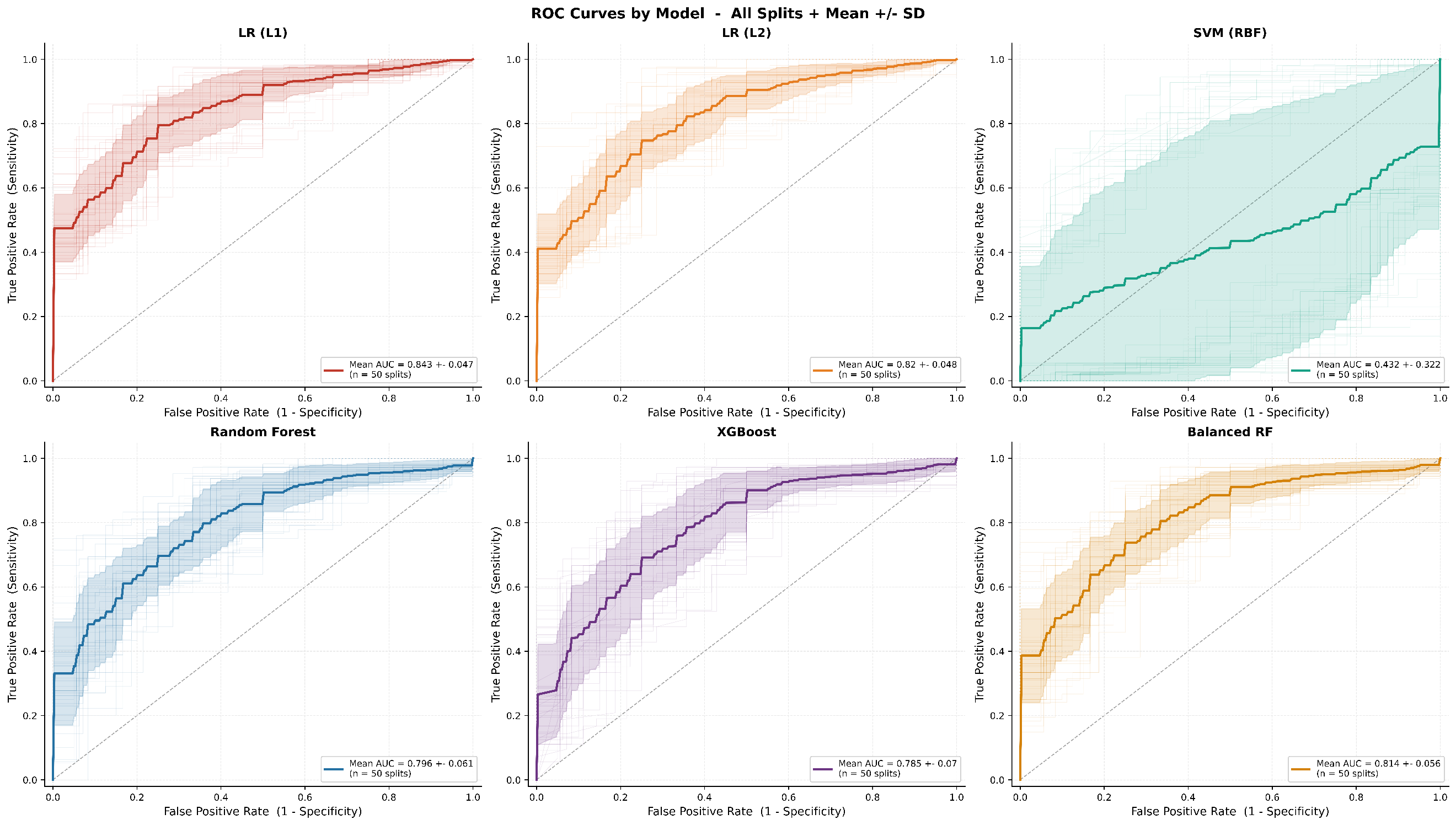


**Figure S3.** ROC curves by different models with 50 splits. Six different ROC curves of classification models in distinguishing between NSSI and non-NSSI. The logistic regression model with L1 has the best model performance with mean AUC of 0.843, while support vector model with RBF kernel showed the worst with mean AUC of 0.432.

Abbreviation: LR (L1), logistics regression with L1 regularization; LR (L2), logistics regression with L2 regularization; SVM (RBF), Support Vector Machine with Radial Basis Function Kernel; RF, Random Forest.


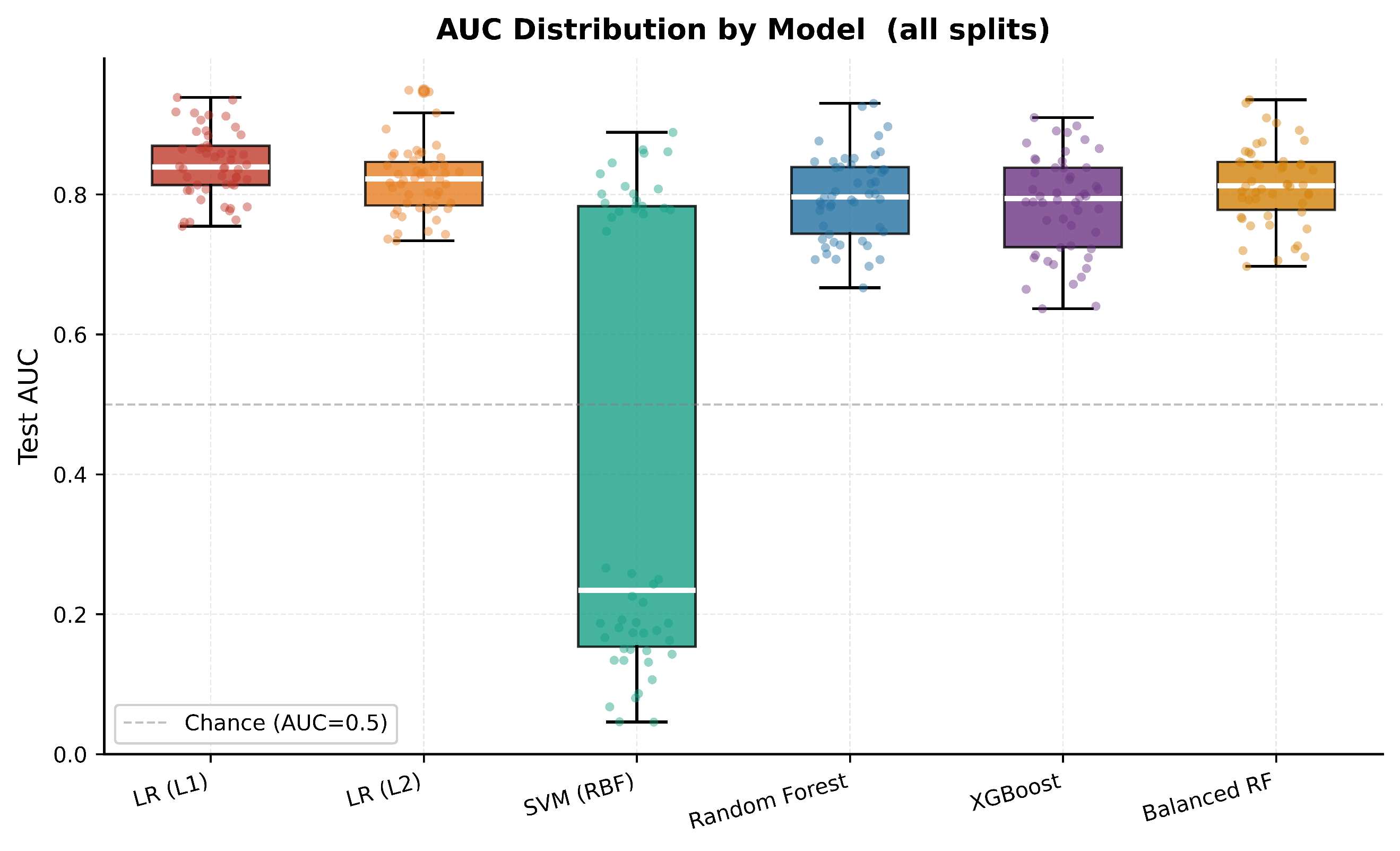


**Figure S4.** Box plot of AUC distribution across models. distribution of test-set ROC-AUC values across all repeated stratified splits and test sizes for each classifier (LR-L1, LR-L2, SVM-RBF, Random Forest, XGBoost, and Balanced Random Forest). The dashed horizontal line marks chance-level performance (AUC = 0.50). Overall, LR with L1 regularization demonstrated the highest and most stable AUC distribution, whereas SVM-RBF showed marked variability and lower performance.

Abbreviation: LR (L1), logistics regression with L1 regularization; LR (L2), logistics regression with L2 regularization; SVM (RBF), Support Vector Machine with Radial Basis Function Kernel; RF, Random Forest.


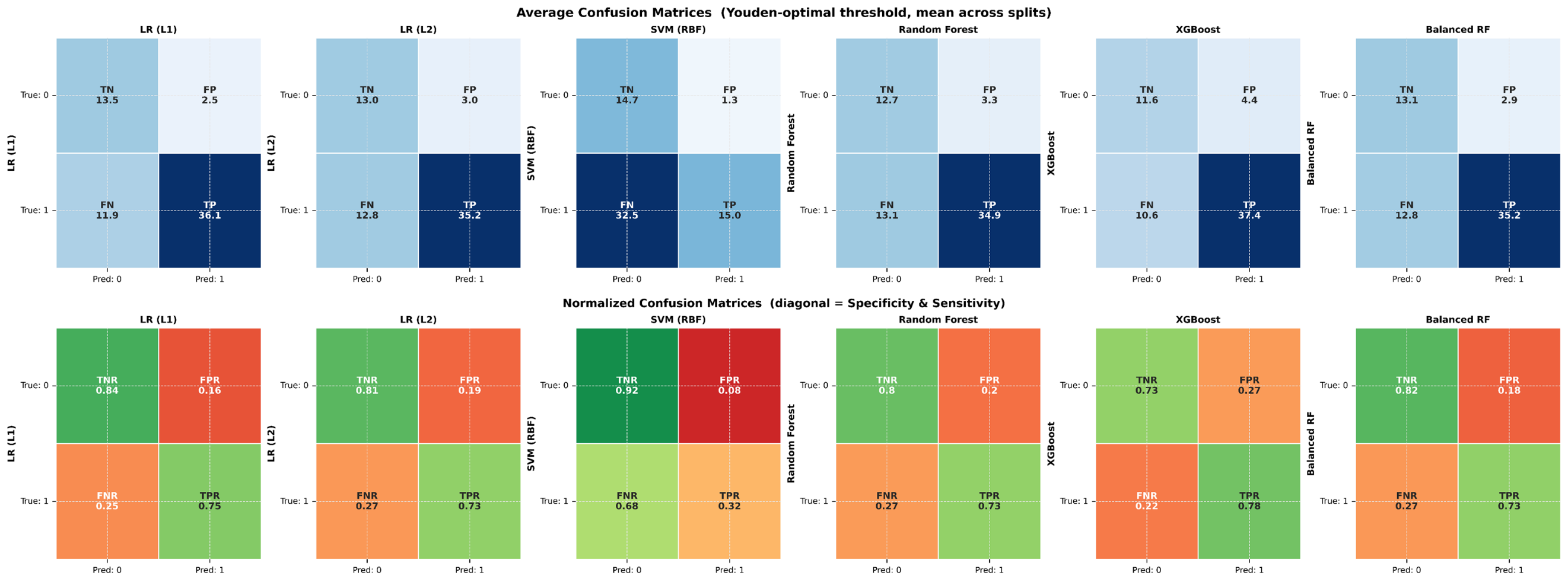


**Figure S5.** Confusion matrices averaged across splits. Top row: Mean confusion matrices across repeated stratified splits for each model, computed on the untouched test sets using a classification threshold determined from training-set out-of-fold predictions (Youden index). Bottom row: Corresponding normalized confusion matrices, with diagonal entries reflecting specificity and sensitivity. Class 1 indicates NSSI and Class 0 indicates non-NSSI.

Abbreviations: LR (L1), logistics regression with L1 regularization; LR (L2), logistics regression with L2 regularization; SVM (RBF), Support Vector Machine with Radial Basis Function Kernel; RF, Random Forest. TN, true negatives; FP, false positives; FN, false negatives; TP, true positives. These matrices summarize model-level error patterns and highlight differences in sensitivity–specificity trade-offs across models.


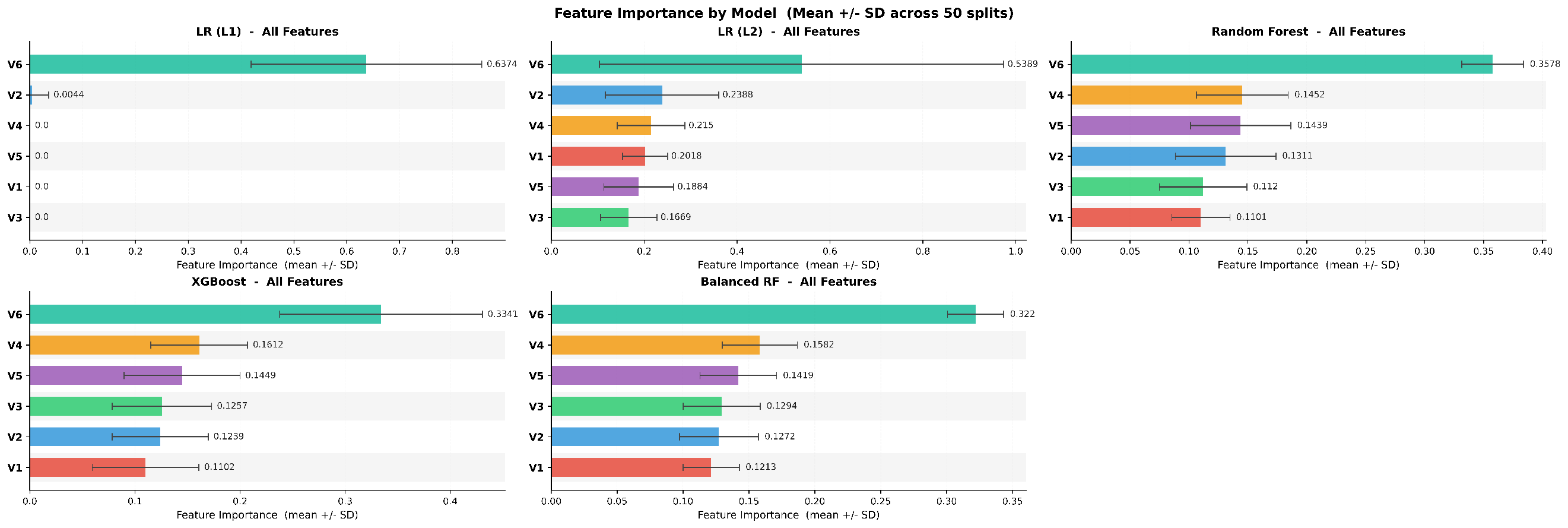


**Figure S6.** Feature importance by model across splits. Feature importance rankings are summarized across all repeated runs for models that provide interpretable importance estimates. For Logistic Regression (L1/L2), importance is defined as the absolute value of standardized model coefficients; for tree-based models (Random Forest, XGBoost, Balanced Random Forest), importance is derived from model-specific impurity/gain-based measures. Bars show mean importance and error bars indicate standard deviation across splits. V1–V6 denote the selected dynamic functional connectivity (dFC) features (connections) used for classification. Results indicate that mean dFC variability consistently contributed strongly to prediction, while ensemble models distributed importance more evenly across features.

Abbreviation : LR (L1), logistics regression with L1 regularization; LR (L2), logistics regression with L2 regularization; SVM (RBF), Support Vector Machine with Radial Basis Function Kernel; RF, Random Forest.V1: right amygdala - left anterior cingulate cortex; V2: right hippocampus - left insula; V3: right insula - right medial orbitofrontal cortex; V4: right insula - left lateral orbitofrontal cortex; V5: left superior prefrontal cortex - left rostral middle prefrontal cortex; V6: mean dFC variability.


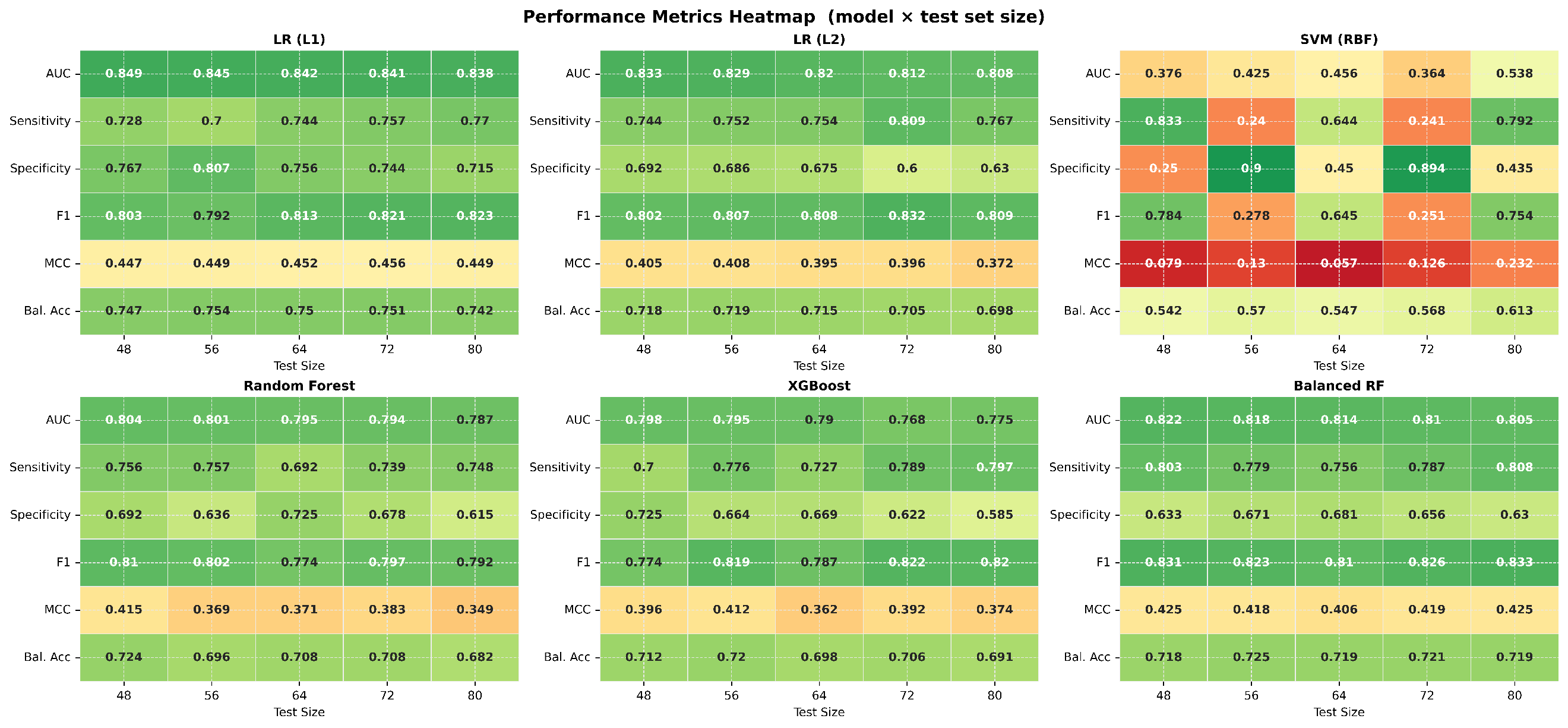


**Figure S7.** Heatmap of performance metrics across models and test sizes. Heatmaps summarize mean classification metrics for each model as a function of hold-out test size (48, 56, 64, 72, and 80 subjects), averaged across repeated stratified splits. Metrics include ROC-AUC, sensitivity, specificity, F1-score, Matthews correlation coefficient (MCC), and balanced accuracy. Warmer colors indicate better performance.

Abbreviation: LR (L1), logistics regression with L1 regularization; LR (L2), logistics regression with L2 regularization; SVM (RBF), Support Vector Machine with Radial Basis Function Kernel; RF, Random Forest.


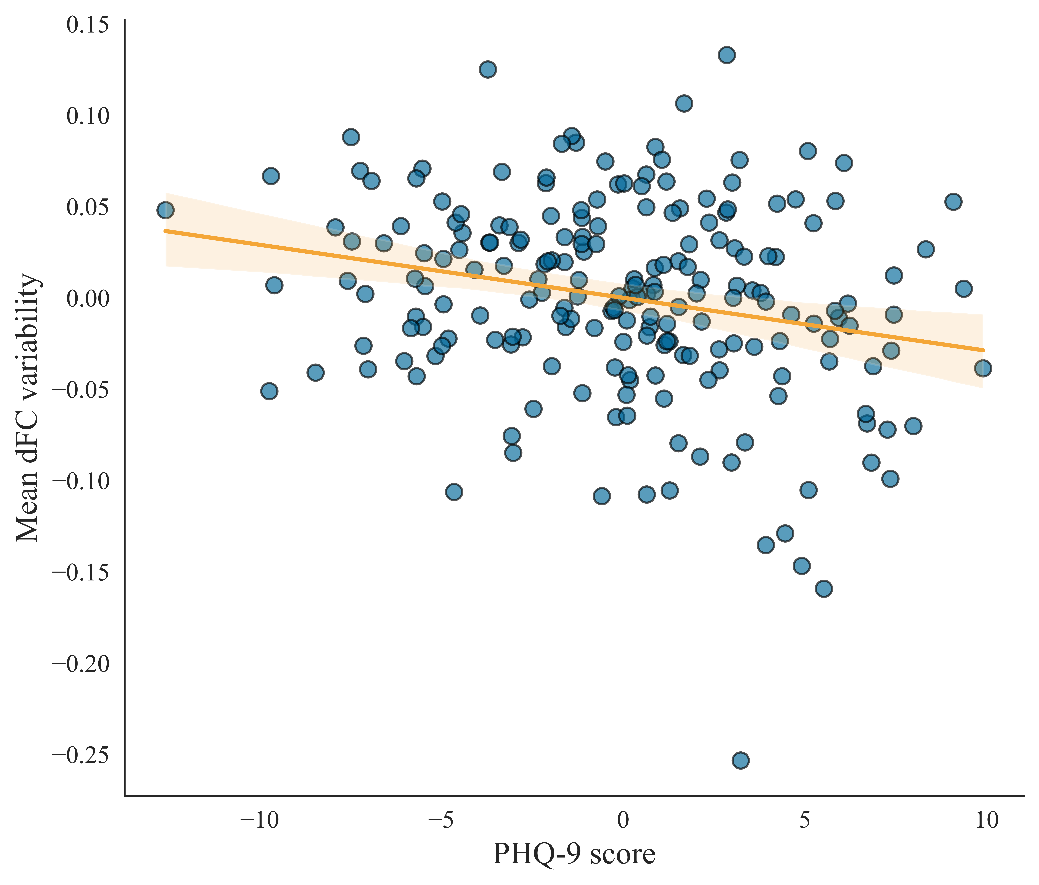


**Figure S8.** Partial correlation between depressive symptoms (PHQ-9 score) and brain dynamic functional connectivity variability. The mean dynamic functional connectivity variability across all group-differentiated connections was significantly correlated with PHQ-9 score (Pearson r = -0.228, p = 0.001) after controlling covariates of age, sex, years of education, family income, GAD-7 score, alcohol use, tobacco use, psychiatric medication, family history of psychiatry and head motion.


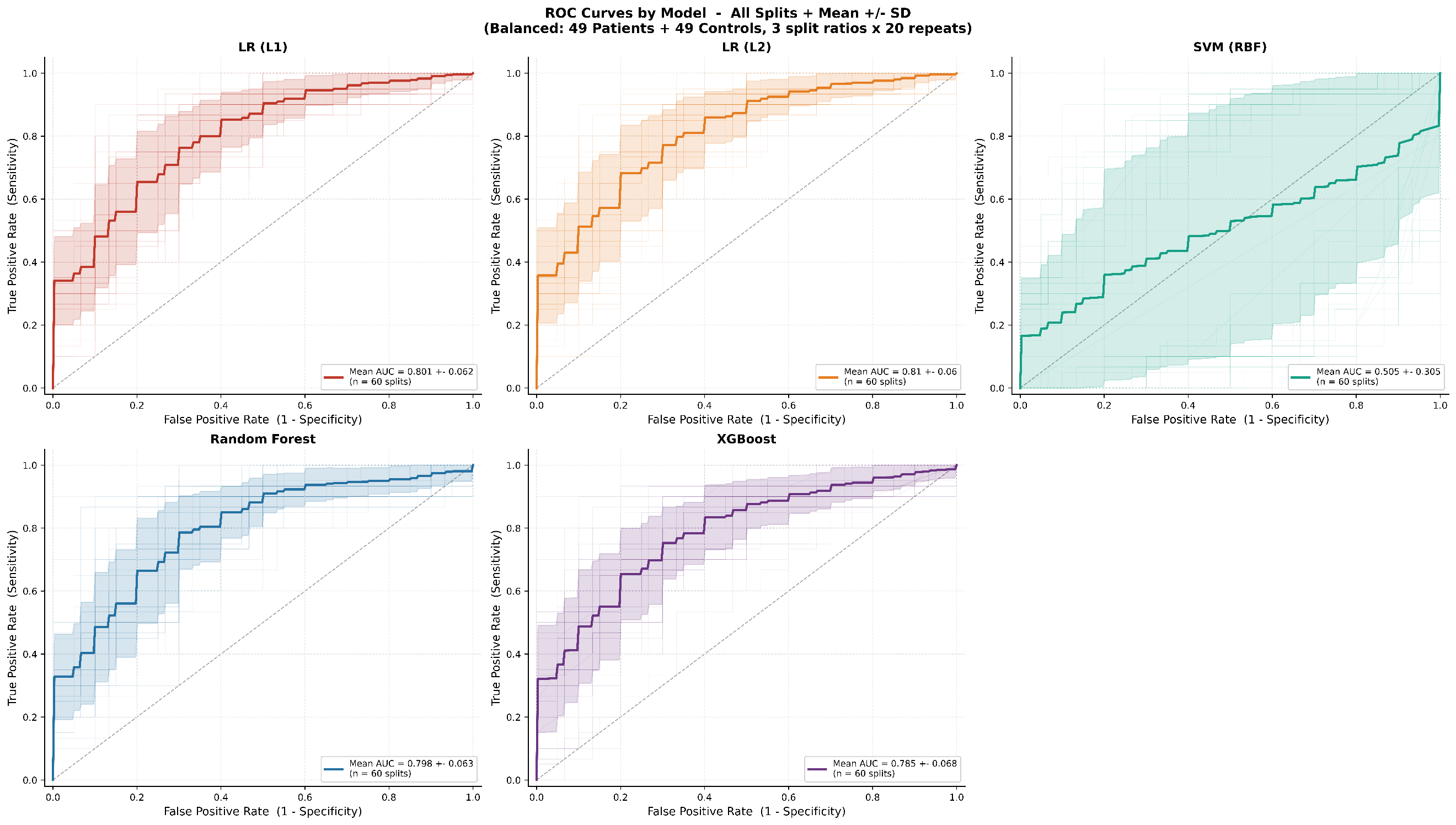


**Figure S9.** ROC curves by different models **in matched group**. Five different ROC curves of classification models in distinguishing between NSSI and non-NSSI. The logistic regression model with L2 has the best model performance with mean AUC of 0.81, while support vector model with RBF kernel showed the worst with mean AUC of 0.505.

Abbreviation: LR (L1), logistics regression with L1 regularization; LR (L2), logistics regression with L2 regularization; SVM (RBF), Support Vector Machine with Radial Basis Function Kernel; RF, Random Forest.


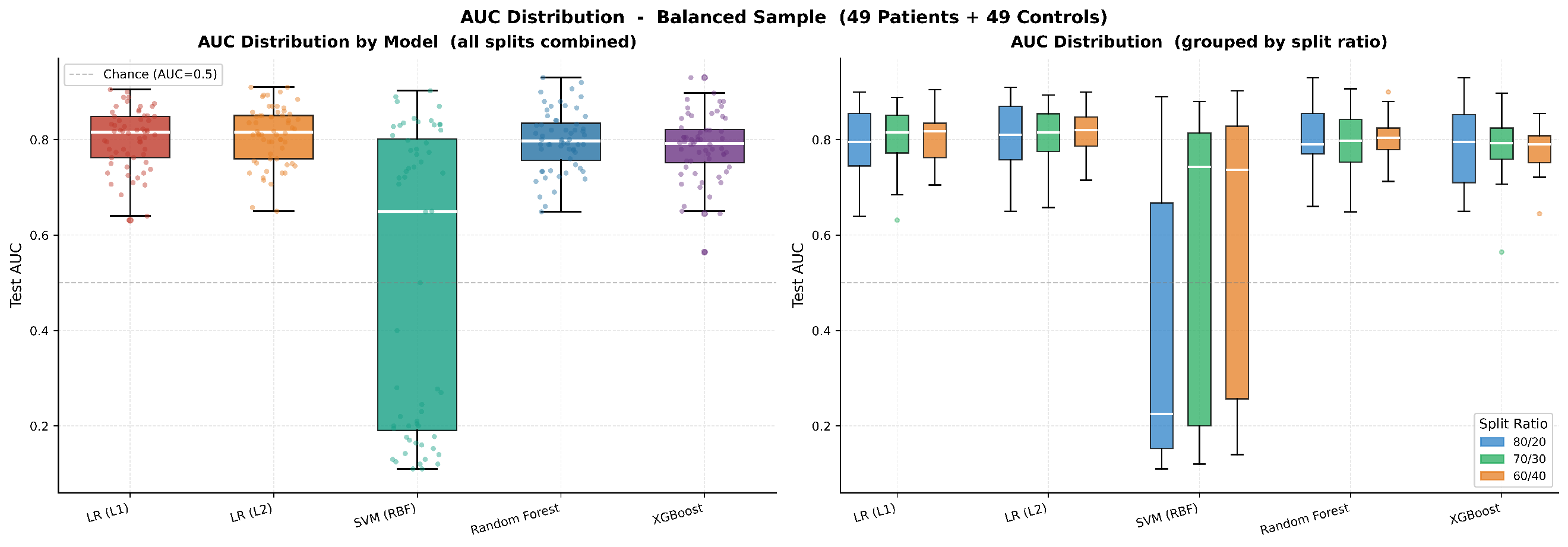


**Figure S10.** Box plot of AUC distribution across models and test size **in matched group**. distribution of test-set ROC-AUC values across all repeated stratified splits and test sizes for each classifier (LR-L1, LR-L2, SVM-RBF, Random Forest, XGBoost, and Balanced Random Forest). The dashed horizontal line marks chance-level performance (AUC = 0.50). Overall, LR with L2 regularization demonstrated the highest and most stable AUC distribution, whereas SVM-RBF showed marked variability and lower performance.

Abbreviation: LR (L1), logistics regression with L1 regularization; LR (L2), logistics regression with L2 regularization; SVM (RBF), Support Vector Machine with Radial Basis Function Kernel; RF, Random Forest.


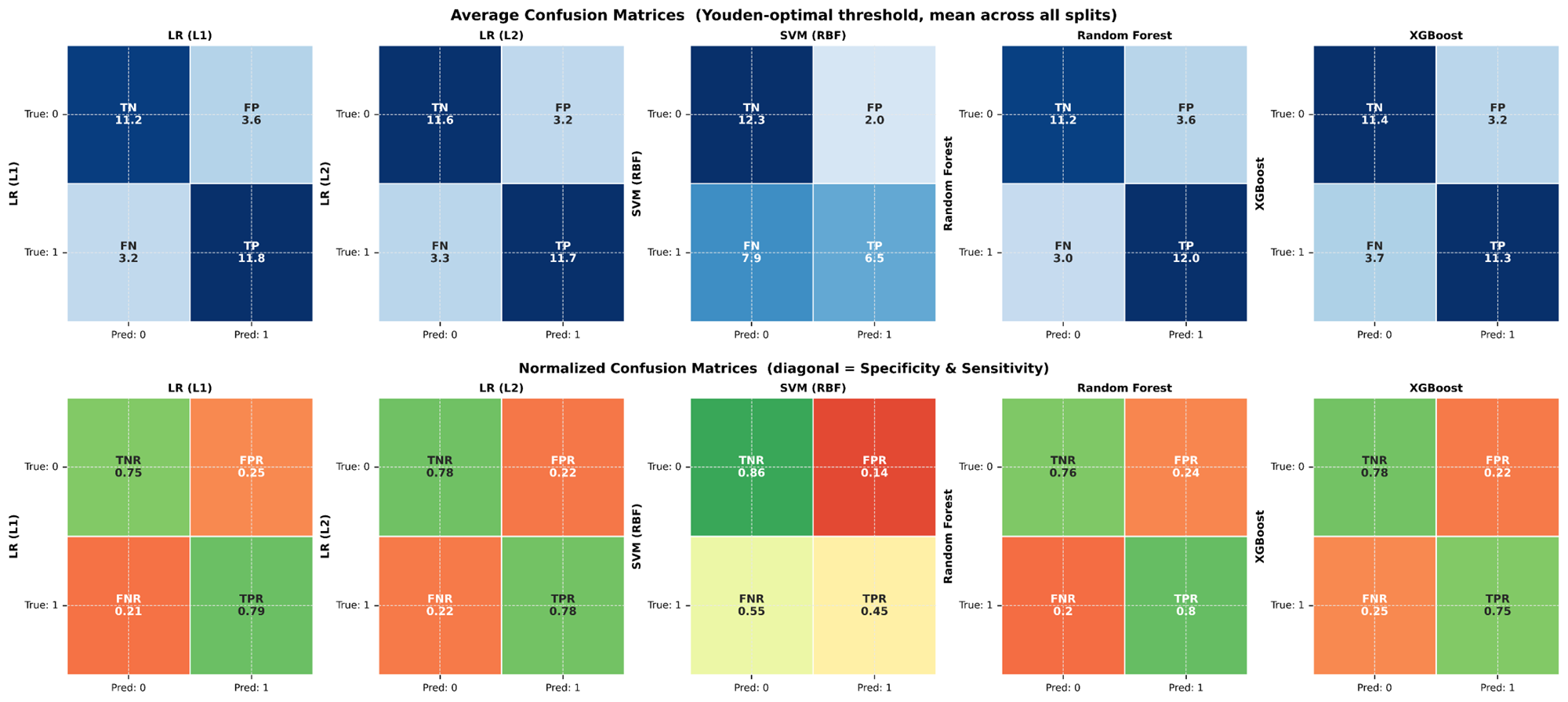


**Figure S11.** Confusion matrices averaged across splits **in matched group**. Top row: Mean confusion matrices across different splits ratio for each model, computed on the test sets using a classification threshold determined from training-set out-of-fold predictions (Youden index). Bottom row: Corresponding normalized confusion matrices, with diagonal entries reflecting specificity and sensitivity. Class 1 indicates NSSI and Class 0 indicates non-NSSI.

Abbreviations: LR (L1), logistics regression with L1 regularization; LR (L2), logistics regression with L2 regularization; SVM (RBF), Support Vector Machine with Radial Basis Function Kernel; RF, Random Forest. TN, true negatives; FP, false positives; FN, false negatives; TP, true positives. These matrices summarize model-level error patterns and highlight differences in sensitivity–specificity trade-offs across models.


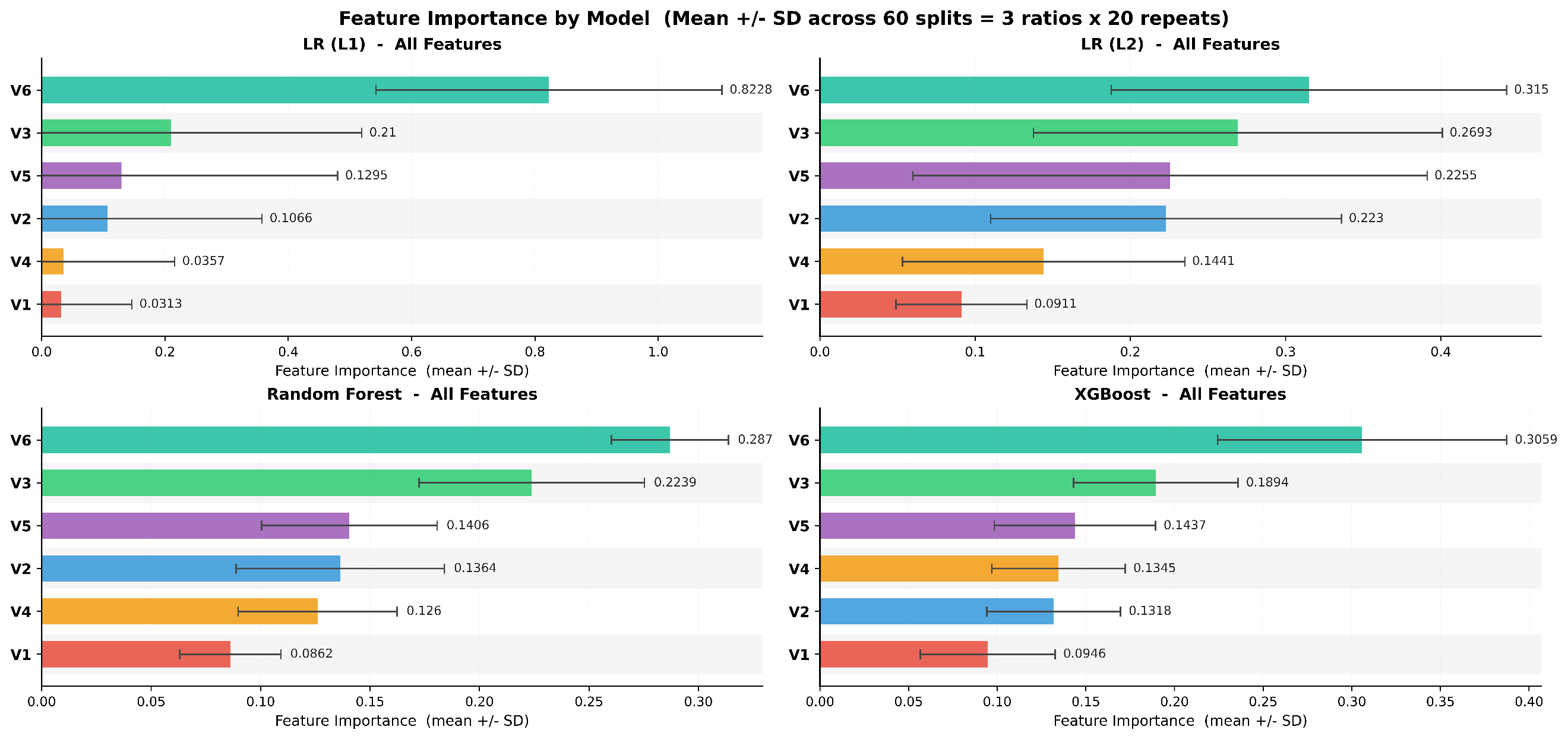


**Figure S12.** Feature importance by model across splits **in matched group**. Feature importance rankings are summarized across all different split ratio runs for models that provide interpretable importance estimates. For Logistic Regression (L1/L2), importance is defined as the absolute value of standardized model coefficients; for tree-based models (Random Forest, XGBoost, Balanced Random Forest), importance is derived from model-specific impurity/gain-based measures. Bars show mean importance and error bars indicate standard deviation across splits. V1–V6 denote the selected dynamic functional connectivity (dFC) features used for classification.

Abbreviation : LR (L1), logistics regression with L1 regularization; LR (L2), logistics regression with L2 regularization; SVM (RBF), Support Vector Machine with Radial Basis Function Kernel; RF, Random Forest.V1: right amygdala - left anterior cingulate cortex; V2: right hippocampus - left insula; V3: right insula - right medial orbitofrontal cortex; V4: right insula - left lateral orbitofrontal cortex; V5: left superior prefrontal cortex - left rostral middle prefrontal cortex; V6: mean dFC variability.


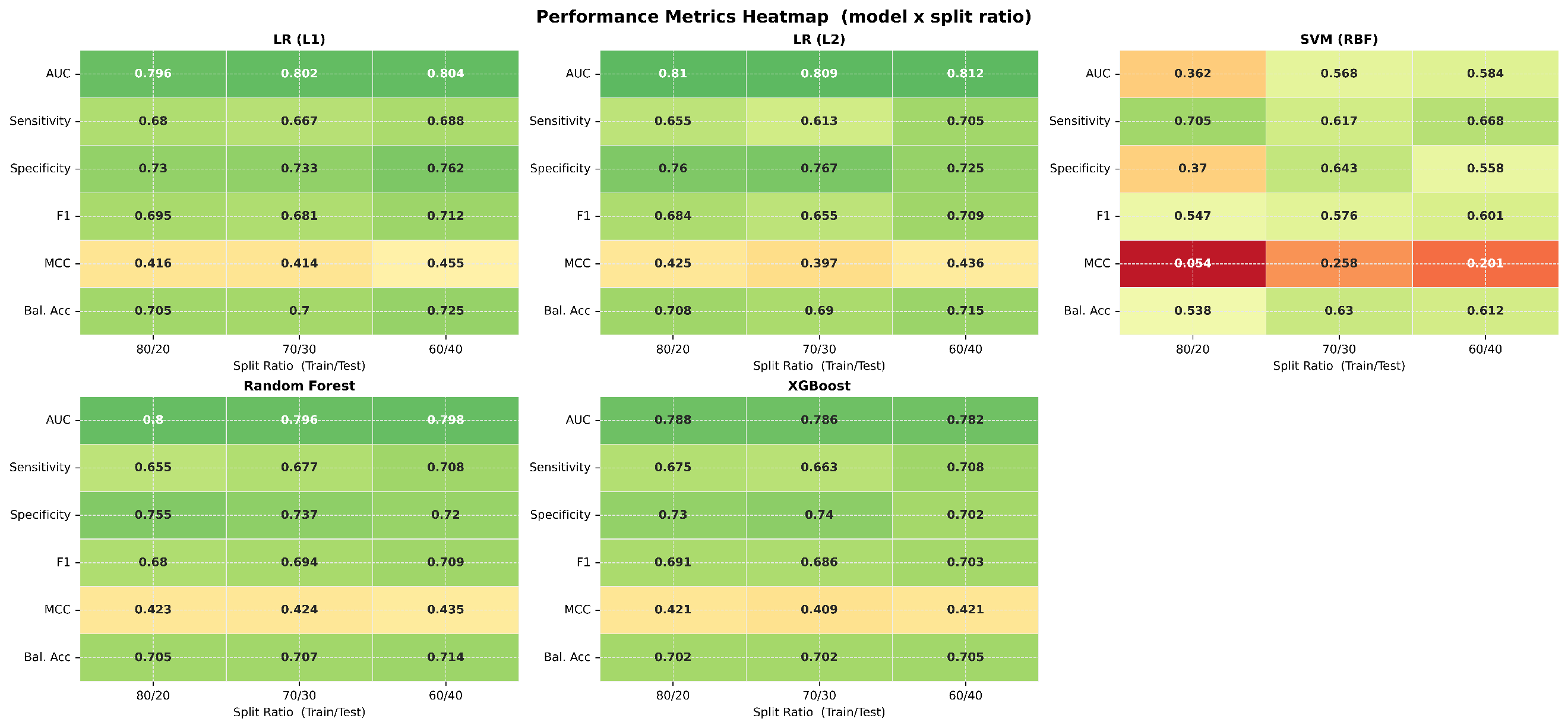


**Figure S13.** Heatmap of performance metrics across models **in matched group**. Heatmaps summarize mean classification metrics for each model across different split ratio (training size/test size, 80/20, 70/30, 60/40). Metrics include ROC-AUC, sensitivity, specificity, F1-score, Matthews correlation coefficient (MCC), and balanced accuracy. Warmer colors indicate better performance.

Abbreviation: LR (L1), logistics regression with L1 regularization; LR (L2), logistics regression with L2 regularization; SVM (RBF), Support Vector Machine with Radial Basis Function Kernel; RF, Random Forest.


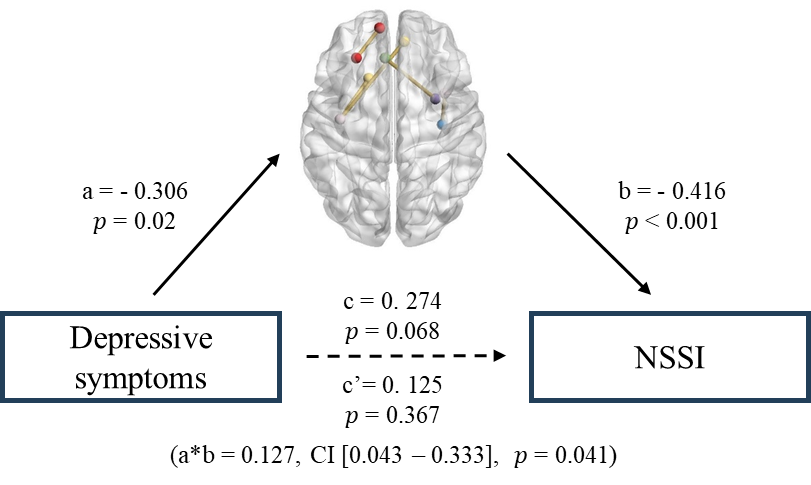


**Figure S14.** Mediation model of severity of depression, NSSI behavior and mean dFC variability **in matched group**. The significant result of mediation analysis showed that the dynamic functional connectivity (dFC) variability links to the severity of depression and NSSI behavior. Path a*b represents an indirect path which is the relationship between depression and NSSI behavior that is mediated by the mean variability of dynamic FC links which were significantly correlated with the depression.

Appendix S1: Methods and Results

Exclusion of Participants

A total of 30 participants were excluded from the analysis. One participant was removed due to excessive head movement during data acquisition, and 29 were excluded because of incomplete questionnaire data. Among the 29 excluded participants, 16 indicated that they had engaged in NSSI but did not complete the follow-up items specifying the type of NSSI behavior. Another 10 provided incomplete behavioral details (e.g., they selected the type of NSSI behavior but did not report the frequency). The remaining 3 participants were excluded because their data were not collected using the standardized FASM protocol.

Clinical assessment

Functional Assessment of Self-Mutilation (FASM)

The Chinese version of the FASM was used to assess the methods, frequency, and functions of NSSI over the past 12 months(Ferrara, Terrinoni, & Williams, 2012). In detail, participants were asked if they have engaged in any of the 10 listed self-harm behaviors (i.e., hitting, head banging, stabbing, pinching, scratching, biting, burning, and cutting) in the past year and the frequency of this behavior. Thereafter, participants who answered ‘yes’ and reported no suicidal intent during these behaviors were classified as the NSSI group and those who answered ‘no’ were classified as the non-NSSI group. The Chinese version of FASM has satisfactory reliability and validity (Tang et al., 2016).

Patient Health Questionnaire-9 (PHQ-9)

The PHQ-9 is a self-evaluation scale that aims to screen one’s depressive symptoms and their severity (Kroenke, Spitzer, & Williams, 2001). The PHQ-9 investigates how often the participant has been bothered by nine different symptoms of depression during the last two weeks, with scores from 0 (not at all) to 3 (nearly every day) on all the questions. Higher scores are indicative of more severe depression (0-4 = minimal or none, 5-9 = mild, 10-14 = moderate, 15-19 = moderately severe, 20 or more = severe depression). The Chinese version of PHQ-9 has been widely applied with good reliability and validity (Zhang et al., 2013). In the current study, the Cronbach's alpha coefficient of the scale is 0.846.

Generalized Anxiety Disorder Scale 7-item (GAD-7)

The GAD-7 is a self-report instrument designed to assess one’s anxiety symptoms and their severity using 7 distinct issues (Spitzer, Kroenke, Williams, & Löwe, 2006). Each item requires respondents to rate the frequency of experiencing anxious symptoms during the past two weeks, with a score of 0 (not at all) to 3 (nearly every day). Higher total scores are indicative of more severe generalized anxiety (0-4 = minimal or none, 5-9 = mild, 10-14 = moderate, 15 or more = severe anxiety). This measurement is widely used in China with good reliability and validity (Sun, Liang, Chi, & Chen, 2021). The Cronbach's alpha coefficient in our research is 0.891.

MRI data acquisition

MRI data were obtained using a 3.0 Tesla scanner MRI (Prisma, Siemens, Germany). High-resolution structural images were acquired with a standard FSPGR T1-weighted sequence using the following parameters: TR = 2530 ms, TE = 3.39 ms, flip angle = 7⁰; field of view [FOV] = 256mm2; 1 mm3 resolution; 192 slices.

The functional images were acquired using an echo-planar imaging (EPI) sequence with the following parameters: TR = 2000ms; TE = 30ms; flip angle = 90⁰; 2 mm3 resolution; FOV = 220mm2; matrix = 64 x 64; 33 axial slices. The scan lasted for 480 s and 240 volumes were acquired.

fMRI data pre-processing

The functional images were preprocessed by Analysis of Functional Neuro Images software (AFNI v 23.1.00; (Cox & Hyde, 1997)). First, the initial 3 functional volumes were removed to stabilize image intensity, followed by slice-time correcting to register each volume to the minimum outlier scan. Then head motion correction was performed using the tool available in AFNI. Subsequently, the functional images were registered to anatomical scan via AFNI’s nonlinear registration tool, following the standardization of anatomical images to the MNI 152 atlas space. Finally, AFNI’s 3dFWHMx was used to smooth each volume to a Gaussian full-width at half-maximum (FWHM) of 6mm. Functional images were band-pass filtered (0.01 < f < 0.1 Hz) for subsequent regression analysis.

To reduce the effects of motion and other brain structures on BOLD correlation, signals from ventricles and white matter masks as well as six motion parameters and their first temporal derivatives were regressed out. Framewise displacement (FD), a volume-by-volume measure of motion was calculated. Time points with FD exceeding 0.3 mm were censored. Participants with a mean head motion exceeding 0.2 mm across all time points were excluded from this analysis. Following the preprocessing steps, one participant was excluded due to excessive motion artifacts.

Classification analysis

Methods

We implemented a supervised machine learning framework to discriminate between adolescents with major depressive disorder (MDD) who engaged in non-suicidal self-injury (NSSI; n = 154) and those without NSSI (n = 50). Classification was performed using imaging-derived features. To ensure a comprehensive evaluation, we compared six classifiers widely utilized in neuroimaging research: (1) Logistic Regression with L1 regularization (LR-L1), (2) Logistic Regression with L2 regularization (LR-L2), (3) Support Vector Machine with a radial basis function (SVM-RBF) kernel, (4) Random Forest (RF), (5) Extreme Gradient Boosting (XGBoost), and (6) Balanced Random Forest. All classifiers were integrated into a standardized preprocessing and resampling pipeline to facilitate rigorous model comparison.

To account for the imbalanced class distribution and ensure robust performance estimation while preventing data leakage, we employed a repeated stratified train–test splitting procedure. To assess model stability across varying data proportions, we systematically varied the test set size (N = 48, 56, 64, 72, 80), while maintaining the original class proportions (stratification) in both sets. For each test size configuration, the data were randomly partitioned 10 times using different random seeds. The test sets were strictly held out and reserved exclusively for final model evaluation, while all preprocessing, hyperparameter tuning, and threshold selection were conducted solely within the training data.

Preprocessing was embedded within a pipeline-based workflow to ensure a strict separation between training and testing information. Missing values were imputed using a median strategy derived exclusively from the training set. Feature scaling was model-dependent: features were normalized using a Robust Scaler for scale-sensitive models (Logistic Regression and SVM), while tree-based models (RF, XGBoost, and Balanced RF) were trained on unscaled data.

To mitigate the impact of class imbalance, Synthetic Minority Oversampling Technique (SMOTE) (Chawla, Bowyer, Hall, & Kegelmeyer, 2002) was incorporated as a tunable pipeline component, applied only to the training data. To ensure a rigorous and leakage-free implementation, the SMOTE procedure was executed within each training fold through a sequential four-step process: first, for each minority class sample (non-NSSI), its *k*-nearest neighbors were identified within the training feature space; second, synthetic samples were generated by randomly interpolating between the original sample and a selected neighbor; third, the number of neighbors (3 or 5) was dynamically adjusted to match the minority class size of each specific fold to maintain mathematical validity; and finally, this synthesis was restricted exclusively to the training set, ensuring that the validation and held-out test sets remained entirely composed of original, non-synthetic clinical data.

Hyperparameter tuning was conducted using a two-stage randomized search procedure nested entirely within the training data. In Stage 1, a coarse hyperparameter search was performed using stratified 5-fold cross-validation. For each model, a predefined hyperparameter space was sampled using randomized search, and model performance was evaluated using the area under the receiver operating characteristic curve (ROC-AUC) as the optimization metric. In Stage 2, the best-performing configuration from Stage 1 was further refined using a repeated stratified 5-fold cross-validation scheme. Specifically, 5-fold stratified cross-validation was repeated five times with different fold partitions, resulting in a total of 25 cross-validation evaluations per hyperparameter configuration. A narrower hyperparameter search space was used at this stage, again optimizing ROC-AUC. This two-stage strategy balanced computational efficiency with robust hyperparameter estimation.

To convert probabilistic model outputs into binary predictions, classification thresholds were determined exclusively from the training data. After hyperparameter optimization, out-of-fold predicted probabilities for the positive class were obtained for each training sample using stratified 5-fold cross-validation, ensuring that each sample was predicted exactly once by a model not trained on that sample. The optimal decision threshold was selected by maximizing the Youden index computed from these out-of-fold predictions. This threshold was then fixed and applied to the independent test set. At no point were test-set labels or predictions used to determine the classification threshold.

Test-set performance was quantified using ROC-AUC, precision–recall AUC, balanced accuracy, sensitivity, and specificity. Feature importance was computed in order to find the most important connection. For linear models, feature importance was quantified using the absolute value of model coefficients. For tree-based models, feature importance scores were extracted from model-derived impurity-based measures. Feature importance estimates were aggregated across repeated splits to assess their stability.

Results

To evaluate the robustness of classification performance under substantial class imbalance (NSSI = 154 vs. non-NSSI = 50), we conducted a systematic sensitivity analysis by varying the hold-out test size (48, 56, 64, 72, and 80 subjects) and performing 10 independent stratified random splits per test size (50 runs per model). Six classification models were compared under an identical preprocessing and validation framework: Logistic Regression (L1 and L2), SVM-RBF, Random Forest, XGBoost, and Balanced Random Forest. Performance was summarized using imbalance-robust metrics, including ROC-AUC, PR-AUC, balanced accuracy, sensitivity, specificity, F1-score and MCC.

Across all test sizes and splits, Logistic Regression with L1 regularization showed the strongest overall discriminative performance (mean ROC-AUC = 0.8428, mean ROC-AUC SD = 0.0497; mean PR-AUC = 0.9440; mean balanced accuracy = 0.7488; mean sensitivity = 0.7398; mean specificity = 0.7579). Balanced Random Forest also performed consistently well (mean ROC-AUC = 0.8139, mean ROC-AUC SD = 0.0589; mean PR-AUC = 0.9306; mean balanced accuracy = 0.7205), with a tendency toward higher sensitivity (mean = 0.7866) but comparatively lower specificity (mean = 0.6543). Logistic Regression (L2) achieved comparable performance (mean ROC-AUC = 0.8201, mean SD = 0.0478; mean PR-AUC = 0.9337). Random Forest and XGBoost yielded slightly lower but still stable performance overall (Random Forest: mean ROC-AUC = 0.7962, mean SD = 0.0632; XGBoost: mean ROC-AUC = 0.7853, mean SD = 0.0720). In contrast, SVM-RBF exhibited markedly lower and unstable performance across splits (mean ROC-AUC = 0.4318, SD = 0.3328), indicating poor generalization in this dataset.

In addition to aggregated performance, we report the best-performing runs for the top two models to provide an interpretable reference point. The best L1 logistic regression run (test size = 56) achieved an ROC-AUC of 0.9388 (bootstrap 95% CI: 0.8609–0.9887), PR-AUC of 0.9803, accuracy of 0.8571, balanced accuracy of 0.8810, sensitivity of 0.8333, and specificity of 0.9286. The corresponding confusion matrix was TN = 13, FP = 1, FN = 7, TP = 35, indicating minimal misclassification. The best balanced Random Forest run (test size = 56) achieved an ROC-AUC of 0.9354 (bootstrap 95% CI: 0.8636–0.9886), PR-AUC of 0.9782, accuracy of 0.8571, balanced accuracy of 0.8333, sensitivity of 0.8810, and specificity of 0.7857, with a confusion matrix of TN = 11, FP = 3, FN = 5, TP = 37.

Feature importance analyses further characterized how the selected dFC features contributed to classification. For L1 logistic regression, importance was derived from the absolute value of the model coefficients aggregated across runs, revealing a highly sparse pattern consistent with L1 regularization: mean dFC variability dominated the importance ranking (mean importance = 0.6374 ± 0.2182, n = 50), whereas the remaining features contributed minimally (e.g., connection of R hippocampus – L insula mean = 0.0044 ± 0.0309; others near zero). In contrast, Balanced Random Forest showed a more distributed attribution across features based on impurity-based importance, with mean dFC variability remaining the top contributor (mean = 0.3220 ± 0.0213, n = 50), followed by R insula – L lateral OFC (0.1582 ± 0.0286), L superior PFC – L rostral middle PFC (0.1419 ± 0.0290), R insula – R medial OFC (0.1294 ± 0.0292), and R hippocampus – L insula (0.1272 ± 0.0299), indicating that classification performance was supported by a combination of features rather than a single connection alone in the ensemble model.

For visualization and transparency, we provide model-wise ROC curves in Figure S3, AUC distributions across test sizes and repeated splits in Figure S4, representative confusion matrices in Figure S5, summarized feature-importance rankings in Figure S6, and a heatmap overview of key performance metrics across models and test sizes in Figure S7.

Appendix S2: Static functional connectivity (FC) analysis

Static functional connectivity (FC) analysis between two groups

Static FC was examined using a ROI-based approach with 55 ROIs within the fronto-limbic network in MATLAB. Briefly, the BOLD signal of each ROI was extracted for each participant. A 55x55 correlation matrix for each participant was constructed by calculating Pearson’s correlation coefficients between 55 ROIs. After that, Fisher’s r-to-z transformation was used to normalize the correlation matrix. Finally, an independent two-sample t-test (two-tailed) was performed on the correlation functional matrix of two groups. The False discovery rate (FDR) correction was applied for multiple comparison corrections with q < 0.05.

204 participants were included in this analysis. The results of this analysis showed that no connections survived after the FDR correction. Table S3 presents the top 5 connections with the smallest uncorrected p-values.

Appendix S3: Matched group analysis

Matched group analysis

Demographics and clinical characteristics

To test the robustness of the results, 98 participants with age and sex-matched were selected using SPSS v26.0 with the function of case-control analysis. The variables matched on sex and age with match tolerances 0 and 1 respectively. As a result, 49 adolescents with NSSI and 49 adolescents without NSSI revealed as matched group sample. The demographics and clinical characteristics are shown in Table S4. All variables were tested by a one-sample Kolmogorov-Smirnov test to check whether data were normally distributed. For continuous variables, such as age, years of education, family income, and head motion, the independent t-test was used and the Mann-Whitney U test was used for PHQ-9 and GAD-7. For sex, we used the chi-square test. The results of demographics and clinical characteristics are shown in Table S4.

Group difference in dFC variability values

In matched group analysis, the connections that showed significant differences in the unbalanced group exhibited significant alteration between the two groups as well (Table S5). Similar to the main research, the dFC variability of all connections in the NSSI group is lower than in the non-NSSI group. Specifically, right amygdala- left ACC (t = -2.373, p = 0.02), right hippocampus- left insula (t = -3.657, p <0.001), right insula- right medial OFC (t = -4.447, p <0.001), right insula-left lateral OFC (t = -2.995, p = 0.004), left superior FC – left rostral middle FC (t = -3.620, p <0.001), and mean dFC variability (t = -5.778, p <0.001).

Classification analysis

We extracted dFC variability of the connections that showed significant group differences in matched two groups to construct classification models. In the matched group classification analysis, we conducted a split-ratio sensitivity analysis to assess model stability across three different test-set proportions: 20%, 30%, and 40% given the relatively small size of sample. To ensure the robustness of our performance estimates, each split configuration was repeated 20 times using independent random seeds, and the results were averaged. The models in matched group included (1) Logistic Regression with L1 regularization (LR-L1), (2) Logistic Regression with L2 regularization (LR-L2), (3) Support Vector Machine with a radial basis function (SVM-RBF) kernel, (4) Random Forest (RF), (5) Extreme Gradient Boosting (XGBoost) except of Balanced Random Forest in that this model were more often applied in the imbalanced sample. Hyperparameter tuning was performed using a two-stage randomized search with nested 5-fold stratified cross-validation which was same as before. Crucially, unlike imbalanced workflows, we maintained a uniform cost function (i.e., no class weighting), as the prior probability for each class was inherently equal. In addition, all synthetic oversampling techniques (SMOTE) were excluded. Instead, Tomek Links was optionally employed as a cleaning step to remove ambiguous boundary samples and improve the separability of the feature space.

In matched group classification analysis, the regularized models and ensemble methods demonstrated competitive diagnostic performance. At the 60/40 split ratio, the Logistic Regression with LR-L2 achieved the highest mean AUC of 0.8115 ± 0.049. the LR-L1 and RF model was close to the LR-L2, which yielded mean AUC of 0.8044 ± 0.05 and 0.7980 ± 0.051, respectively. The XGBoost classifier also showed robust performance with a mean AUC of 0.7823 ± 0.051. In contrast, the SVM-RBF model still exhibited lower and more volatile performance with mean AUC of 0.5837 ± 0.292. Beyond AUC, the LR-L2 achieved a balanced accuracy of 71.5%, with a mean sensitivity of 70.5% and Specificity of 72.5%. The RF model displayed a comparable balanced profile with Balanced Accuracy of 71.4%, showing a slightly higher Specificity of 72.0% but similar Sensitivity of 70.8%.

For the sensitivity and model stability analysis, the mean AUC for Random Forest remained remarkably consistent, ranging from 0.7958 in 70/30 split to 0.7995 in 80/20 split. Similarly, LR-L1 model maintained high discriminative power across all configurations, with AUC values consistently exceeding 0.79.

For the feature importance analysis, across the different models, mean dFC variability and connection of right insula – right medial orbitofrontal cortex were consistently identified as the top two contributors to the decision boundary. Specifically, in the RF model, mean dFC variability had a mean importance of 0.287, while in XGBoost, it accounted for 0.306 of the gain.

For visualization and transparency, we provide model-wise ROC curves in Figure S9, AUC distributions across split ratios in Figure S10, representative confusion matrices in Figure S11, summarized feature-importance rankings in Figure S12, and a heatmap overview of key performance metrics across models in Figure S13.

Correlation analysis and mediation analysis

To further examine the robustness of the mediation findings, we conducted an additional mediation analysis in the age- and sex-matched subsample. The correlation analysis results are shown in Table S6 Same to the main analysis that the mean value of dFC variability exhibited the highest correlation with PHQ-9 scores (r = -0.236, p < 0.05), other brain connections that showed significant correlation are right insula-right medial OFC (r = -0.265, $p$ = 0.014), right insula-left lateral OFC (r = -0.221, $p$ = 0.041), left superior frontal cortex - left rostral middle frontal cortex (r = -0.254, $p$ = 0.018). After bonferroni correction, only mean dFC variability survive.

We then tested whether mean dFC variability mediated the association between depressive symptom severity and NSSI engagement in the matched subsample. The mediation model was estimated in Mplus using the WLSMV estimator with a probit link because NSSI engagement was treated as a categorical outcome. The model adjusted for the same set of covariates as the main analysis, including age, sex, years of education, family income, anxiety severity, alcohol use, tobacco use, psychiatric medication status, family history of psychiatric disorders, and head motion. The indirect effect was tested using bias-corrected bootstrap confidence intervals, with 10,000 bootstrap resamples requested.

The matched-group mediation analysis showed a pattern generally consistent with the primary analysis in **Figure S14.** The indirect effect of depressive symptom severity on NSSI engagement through mean dFC variability was significant (a*b = 0.127, 95% bootstrap CI [0.043, 0.333], p = 0.041. After accounting for mean dFC variability, the direct effect of depressive symptom severity on NSSI engagement was attenuated and was not significant (c′ = 0.125, p = 0.367), suggesting that the association between depressive symptoms and NSSI was mainly explained by the indirect pathway through reduced mean dFC variability.

In the matched group mediation model, the total effect of PHQ-9 score on NSSI engagement was positive but did not reach statistical significance in the standardized model (c = 0.274, CI [0.008 – 0.599], SE = 0.150, p = 0

.068). As an additional sensitivity analysis using a conventional binary logistic regression model with the same set of covariates, PHQ-9 score was significantly associated with NSSI engagement (β = 0.151, SE = 0.066, p = 0.023). Thus, although the statistical significance of the total effect varied slightly across link functions and estimation frameworks, the direction of the association was consistent across models.

Taken together, the matched-subsample analyses supported the robustness of the primary mediation findings. The direction of the associations among depressive symptom severity, mean dFC variability, and NSSI engagement remained consistent with the main analysis.

Reference

Chawla, N. V., Bowyer, K. W., Hall, L. O., & Kegelmeyer, W. P. (2002). SMOTE: synthetic minority over-sampling technique. *Journal of artificial intelligence research, 16*, 321-357.

Ferrara, M., Terrinoni, A., & Williams, R. (2012). Non-suicidal self-injury (Nssi) in adolescent inpatients: assessing personality features and attitude toward death. *Child Adolesc Psychiatry Ment Health, 6*, 12. doi:10.1186/1753-2000-6-12

Kroenke, K., Spitzer, R. L., & Williams, J. B. (2001). The PHQ-9: validity of a brief depression severity measure. *J Gen Intern Med, 16*(9), 606-613. doi:10.1046/j.1525-1497.2001.016009606.x

Spitzer, R. L., Kroenke, K., Williams, J. B., & Löwe, B. (2006). A brief measure for assessing generalized anxiety disorder: the GAD-7. *Arch Intern Med, 166*(10), 1092-1097. doi:10.1001/archinte.166.10.1092

Sun, J., Liang, K., Chi, X., & Chen, S. (2021). Psychometric Properties of the Generalized Anxiety Disorder Scale-7 Item (GAD-7) in a Large Sample of Chinese Adolescents. *Healthcare (Basel), 9*(12). doi:10.3390/healthcare9121709

Tang, J., Yang, W., Ahmed, N. I., Ma, Y., Liu, H. Y., Wang, J. J., . . . Yu, Y. Z. (2016). Stressful Life Events as a Predictor for Nonsuicidal Self-Injury in Southern Chinese Adolescence: A Cross-Sectional Study. *Medicine (Baltimore), 95*(9), e2637. doi:10.1097/md.0000000000002637

Zhang, Y. L., Liang, W., Chen, Z. M., Zhang, H. M., Zhang, J. H., Weng, X. Q., . . . Zhang, Y. L. (2013). Validity and reliability of Patient Health Questionnaire-9 and Patient Health Questionnaire-2 to screen for depression among college students in China. *Asia Pac Psychiatry, 5*(4), 268-275. doi:10.1111/appy.12103
